# Experimental tests of the tertiary transfer effect of intergroup contact: Addressing valence, prototypicality and moderators

**DOI:** 10.1111/bjso.70113

**Published:** 2026-07-08

**Authors:** Ece Aleyna Demirgünes, Jessica Boin, Jasper Van Assche, Giulia Fuochi

**Affiliations:** ^1^ Department of Social and Personality Psychology University of Wuppertal Wuppertal Germany; ^2^ Department FISPPA – Applied Psychology Section University of Padova Padova Italy; ^3^ Center for Social & Cultural Psychology, Université Libre de Bruxelles Bruxelles Belgium; ^4^ Optentia Research Unit North‐West University Vanderbijlpark South Africa

**Keywords:** cognitive liberalization, deprovincialization, intergroup contact, SDO, tertiary transfer effect, vicarious contact

## Abstract

Cognitive liberalization theory posits a tertiary transfer effect (TTE) whereby intergroup contact broadens open‐minded cognitive dispositions. Across two pre‐registered studies, we tested whether vicarious contact with Muslims (among non‐Muslim Italians) influences abstract reasoning, convergent creativity, self‐reported cognitive flexibility, cultural and group deprovincialization and environmental concern, with social dominance orientation (SDO), need for closure (NFC) and curiosity as moderators. Study 1 (*N* = 394) manipulated interaction valence (positive vs. negative) and target (ingroup vs. outgroup) and Study 2 (*N* = 574) manipulated valence and semantic distance (atypical vs. prototypical outgroup members). We hypothesized stronger cognitive gains from intergroup contact, especially with atypical outgroup members. Overall, results provided little support for the main hypotheses or for TTE, aside from targeted effects. In Study 1, positive vicarious interactions increased creativity. Additionally, low‐SDO participants showed reduced abstract reasoning and low‐NFC participants showed increased creativity following positive contact. In Study 2, atypical outgroup contact decreased deprovincialization and creativity, yet high‐SDO participants reported greater cultural deprovincialization after positive contact with atypical outgroup members. These findings suggest a more nuanced and conditional view on TTE: despite evidence for a broad TTE being limited, under specific conditions, vicarious contact could facilitate cognitive liberalization.

## INTRODUCTION

Recently, Hodson et al. (2018) hypothesized that intergroup contact, by exposing individuals to the outgroup's worldviews, could challenge ingroup members' cognitive schemata and stimulate more complex, open‐minded, out‐of‐the‐box thinking; this process was termed ‘cognitive liberalization’ or the tertiary transfer effect (TTE) of contact. The TTE extends beyond intergroup attitudes by ‘promoting deprovincialized thinking, challenging worldviews, and improving problem solving, flexible thinking, and creativity’ (Hodson et al., 2018, p. 524). Meleady et al. (2019) also theorized that TTEs may be most likely when the contacted outgroup member is perceived as atypical (high semantic distance) relative to the group prototype because expectancy‐incongruent exemplars can prompt more systematic information processing and challenge one's schemata, thereby enhancing cognitive flexibility and open‐mindedness.

Only a few studies have examined the TTE of intergroup contact so far, employing a variety of measures to capture cognitive liberalization as outcome variable and focusing on different facets of open‐mindedness: cognitive measures such as cognitive flexibility, and attitudinal measures such as reduced ethnocentrism, lower social dominance orientation (SDO) and higher environmental concern. Environmental concern can be considered a distal socio‐cultural indicator of cognitive liberalization because prior work has linked intergroup contact to environmental concern through egalitarian ideologies, and it is grounded in acceptance of change (see Meleady et al., 2021). Interestingly, some of these cognitive liberalization outcomes are trait‐like variables (e.g., SDO, cognitive flexibility) that are typically studied as antecedents or moderators of the effects of contact, thereby raising doubts on the directionality of the effects when using cross‐sectional data.

Bagci et al. (2019) conducted a cross‐sectional study revealing that, for the Turkish majority, cross‐group friendship with Kurds was linked to lower ethnocentrism and SDO, mediated by more positive attitudes towards Kurds. For the minority group, cross‐group friendship was directly and negatively linked to ethnocentrism and SDO without mediation through outgroup attitudes. For both groups, cross‐group friendships were associated with higher cognitive flexibility, albeit through different mechanisms. Meleady et al. (2020) carried out two cross‐sectional and one longitudinal study to investigate the positive and negative contact experiences of British individuals with Eastern Europeans and Black people. Their cross‐sectional findings showed that positive and negative contact were associated with higher and lower intercultural competence, respectively. In the longitudinal analysis, past positive contact predicted increased intercultural competence over time, whereas past intercultural competence predicted reduced present negative contact. In three cross‐sectional and one longitudinal studies, Meleady et al. (2021) found that intergroup contact was associated with more egalitarian ideologies, such as reduced SDO, leading to an increase in environmentally supportive attitudes and behaviours.

More recently, Jolley et al. (2023) across two cross‐sectional studies found that positive intergroup contact was linked to fewer conspiracy beliefs, which reflect low critical thinking and need for cognitive closure, even when controlling for general prejudice; this result was experimentally replicated with imagined positive contact—compared with an imagined neutral interaction (Jolley et al., 2023). Extending this line of work, Jolley et al. (2025) found that parasocial contact with transgender TikTok creators increased perspective taking among cisgender participants, which in turn was associated with lower transgender conspiracy beliefs, suggesting that media‐based contact can be associated with distal socio‐cognitive outcomes beyond traditional prejudice reduction. Navarro et al. (2023) investigated the TTE of contact within sexual minorities and found that, for gay people, contact with other sexual minorities (i.e. inter‐minority contact) was related to greater cognitive flexibility, which, in turn, was associated with higher well‐being.

Fuochi et al. (2024) conducted a latent profile analysis over a set of variables capturing both cognitive (cognitive flexibility, socio‐cognitive mindfulness, curiosity and low need for structure) and socio‐cultural indices of cognitive liberalization (beliefs supporting societal diversity, low SDO and deprovincialization). The authors found that those participants belonging to the cognitive liberalization profile, that is, with high scores on all cognitive liberalization variables compared to the other profiles portraying cognitive rigidity and anti‐liberalization, reported less negative, and more positive and intimate contact experiences with the outgroup. Moreover, they perceived the outgroup members involved in the positive contact experience as more representative of their outgroup. The authors also addressed the role of semantic distance in TTE. However, contrary to the theoretical predictions of Meleady et al. (2019), they found that people with a cognitive liberalization profile perceived the outgroup members they had positive contact with as more representative of their outgroup, suggesting the need for further tests of prototypicality in the TTE.

In an intensive longitudinal study, Shulman et al. (2025) showed between‐person associations between intergroup contact and higher cognitive flexibility, but no within‐person increases in cognitive flexibility following increases in contact; the authors argued that a stable measure of cognitive skills may not show short‐term fluctuations. Another longitudinal study (Friehs et al., 2025) found robust between‐person associations but no consistent within‐person association between contact and ideology or politics, except for a negative link between negative contact and social‐equality preferences.

### The present research

Despite the growing interest in the TTE of intergroup contact, we identified four missing aspects in this research area: (a) strong experimental designs; (b) a rigorous test of the role of semantic distance; (c) the inclusion of other forms of contact, since all the evidence on TTE regards direct contact, with only one study measuring imagined contact (i.e. Jolley et al., 2023); (d) an exploration of moderators of the TTE. First, experimental designs can test whether contact predicts open‐mindedness and cognitive liberalism, disentangling this relationship from the reverse one, that is, from open‐mindedness to contact, which has received more empirical support (e.g. Turner et al., 2020). Second, the role of semantic distance has been investigated only by Fuochi et al. (2024), with results not supporting the original theory by Meleady et al. (2019).

Third, indirect contact is relevant for the TTE because it encourages direct contact (e.g. Vezzali et al., 2014), it may help to create more structured and replicable experimental manipulations, and it can be employed to overcome geographical and psychological barriers to direct contact. Moreover, some forms of indirect contact may be especially appropriate to foster cognitive liberalization. Indeed, previous studies showed that observing ingroup–outgroup interactions—that is, vicarious contact, which can occur both in person and through the media—was associated with improved intergroup outcomes via learning about the outgroup, identification with the protagonist and narrative engagement (Ortiz & Harwood, 2007; Vezzali et al., 2014). These mechanisms can facilitate cognitive liberalization because they help ingroup members take the perspective of outgroup members, thereby engaging in a cognitive exercise based on complex thinking and stepping outside their own mindset. Fourth, investigating moderators of the TTE is important because cognitive liberalization could exist only for people who are not ‘liberalized’ yet—for example, with high SDO and low openness to diversity experiences—or, on the contrary, could be stronger for people who are already open‐minded.

In order to fill these gaps in cognitive liberalization research, we conducted two pre‐registered experimental studies comparing different types of vicarious interaction and addressing three aims: (Aim 1) to test the effect of positive (compared to negative) intergroup (compared to intragroup) vicarious contact on cognitive liberalization variables; (Aim 2) to test the effect of atypical (compared to prototypical) and positive (compared to negative) vicarious contact with the outgroup on cognitive liberalization variables; (Aim 3) to examine the moderating roles of SDO, need for cognitive closure (NFC; Roets & Van Hiel, 2011) and openness to new experiences and information—as measured by the Joyous Exploration dimension of curiosity (Kashdan et al., 2018), selected because this facet best aligns with the cognitive liberalization hypothesis—on the effects observed when testing Aim 1 and Aim 2. Study 1 tested Aim 1 and Aim 3, whereas Study 2 tested Aim 2 and Aim 3. The outgroup was Muslim people, with vicarious intergroup interactions occurring between non‐Muslim Italian young adults (i.e. the ingroup) and a Muslim young adult. The manipulations consisted of short videos composed of excerpts from a popular television series, selected to mirror the experimental conditions (i.e. group dynamics, valence and prototypicality), while preserving sufficient narrative context to ensure that the depicted interactions were understandable.

Aim 1 specifically assessed the TTE of contact (i.e. intergroup vs. intragroup interactions), also considering its valence. A comparison condition involving interpersonal interactions with ingroup members—rather than a no‐interaction control condition—allowed us to estimate the effect of intergroup contact on cognitive liberalization net of the effects of others' presence, interpersonal engagement and sharing, which are interactional factors that may foster open‐mindedness and other cognitive skills. An active control condition with interpersonal interactions should thus provide a more stringent test of the specific impact of intergroup, as opposed to intragroup, contact. At the same time, we acknowledge that this choice does not allow us to determine whether interactions per se—independent of group membership—would produce changes relative to no interaction. Similarly, Aim 1 and Aim 2 examine the effect of contact valence (positive vs. negative) on cognitive liberalization without including a neutral‐valence control condition. This choice was made to avoid ambiguity and heterogeneity in the subjective interpretations of neutral contact situations, which may be perceived as either positive or negative by participants, and to directly assess the effect of prototypicality across the two opposite‐valence contact conditions.

As dependent variables measuring cognitive liberalization, we employed three performance‐based cognitive indicators (abstract reasoning, creative convergent thinking and creativity), a self‐report measure of cognitive flexibility and three socio‐cultural (cultural deprovincialization, group deprovincialization and environmental concern) measures of cognitive liberalization. Cultural and group deprovincialization were treated as related but distinct facets of deprovincialization (Verkuyten et al., 2022). Group deprovincialization reflects an ingroup‐oriented facet, namely the tendency to place one's own national or cultural worldview into perspective rather than treating it as the only valid frame of reference (Lucarini et al., 2023). Cultural deprovincialization reflects an outgroup‐oriented facet, namely openness, curiosity and acceptance towards other cultures and worldviews (Boin et al., 2020; Lucarini et al., 2023). Consistent with previous findings showing that stable cognitive indicators may not fluctuate over short intervals and following intergroup contact (Shulman et al., 2025), we acknowledge that cognitive skills, especially performance‐based cognitive indicators, may be comparatively less sensitive to short, single‐exposure manipulations compared to socio‐cultural openness indicators, which may be more malleable via intergroup exposure.

Relatedly, we acknowledge that the proposed moderators—that is, SDO, NFC and curiosity—could also be conceptualized as outcomes of cognitive liberalization. However, to distinguish between these two processes, we assessed the dependent variables immediately after the manipulations using shorter scales, whereas the moderators were measured in the final section of the questionnaire, with specific instructions encouraging participants to think of their stable dispositions. Moreover, SDO, NFC and curiosity are theoretically relevant moderators of cognitive liberalization because they capture different forms of openness or resistance to the schema‐challenging information provided by contact (e.g. Meleady et al., 2019). SDO reflects endorsement of hierarchical relations among social groups and may therefore reduce receptivity to egalitarian or deprovincializing implications of contact, although high‐SDO individuals may also have more room to shift following positive or stereotype‐disconfirming contact (Sidanius & Pratto, 1999). NFC reflects a preference for certainty and discomfort with ambiguity, which may make atypical or expectancy‐incongruent outgroup information more difficult to process, but may also make structured positive contact especially consequential (Roets & Van Hiel, 2011; Webster & Kruglanski, 1994). Finally, Joyous Exploration reflects intrinsic interest in novelty and learning and may therefore facilitate engagement with unfamiliar perspectives, although highly curious individuals may already be comparatively open to such experiences (Kashdan et al., 2018). Because these competing possibilities were plausible, we preregistered moderation by SDO, NFC and curiosity without specifying directional hypotheses.

Based on the cognitive liberalization hypothesis and prior evidence (e.g. Meleady et al., 2019, 2021), we hypothesized (in the pre‐registrations) that (1) vicarious contact with the outgroup (vs. the ingroup) would increase cognitive liberalization regardless of contact valence (H1), as both positive and negative contact are cognitively engaging experiences, differing only in their emotional tone—pleasant versus unpleasant (e.g. Crisp & Turner, 2011); (2) vicarious contact with an atypical (vs. prototypical) outgroup member would increase cognitive liberalization, with stronger effects when contact is positive, because with positive contact group membership is less salient (Paolini et al., 2010) (H2); (3) the TTEs of intergroup contact would be moderated by SDO, NFC and curiosity (Joyous Exploration).

Analyses were performed with R (R Core Team, 2024), especially using the packages *emmeans* (Lenth, 2024), *effects* (Fox & Weisberg, 2019), and *reghelper* (Hughes, 2022).

## STUDY 1

### Method

The Psychological Research Ethics Committee of the University of Padova approved the procedures of this study, protocol #5133. Data, R script, video description and pre‐registration are available on OSF (https://doi.org/10.17605/OSF.IO/34H2E).

#### Participants

Participants were recruited through acquaintances of students enrolled in a social psychology course, and they received course credit for their participation. We conducted a priori power analysis using G*Power (Faul et al., 2007), by setting 80% power, .05 significance level and small‐to‐medium effect size (*f* = 0.15), consistent with effect sizes from previous research investigating cognitive liberalization (e.g. Meleady et al., 2021; Navarro et al., 2023): the minimum sample size was 351. We recruited 503 participants. However, participants, who self‐identified as Muslim (*N* = 2), did not provide consent (*N* = 6), made more than one error across four attention manipulation check items about video content (see Supplementary Materials for details; *N* = 38), spent more than 350 s (video duration: around 230 s) on the video page (*N* = 29), or took an excessively long time (more than 1 h) to complete the questionnaire (*N* = 34) were excluded from the analysis. The 350‐s cut‐off was set to allow up to 50% additional time beyond the video's duration, based on typical participant behaviour (e.g. brief pauses, minor distractions) and potential technological delays. After applying these exclusions, the final sample size was *N* = 394.^1^ We conducted a sensitivity analysis: given our final sample of *N* = 394 (α = .05, 1 − β = .80), the design had adequate power to detect effects of *f* = 0.14 (*f* ^2^ = 0.02, *R* ^2^ = 0.02), that is, in the small‐to‐medium range.

Participants' ages ranged between 18 and 85 years (*M* = 31.43, *SD* = 14.71). As for gender, 231 (59%) self‐identified as woman, 156 (40%) self‐identified as man and 7 (2%) as ‘other’ or non‐binary. Their education levels were varied: the highest education level attained was secondary school for 6%, a high school diploma for 55%, a bachelor's degree for 19% and higher degrees (master's or PhD degrees) for 20% of the sample.

#### Procedure

This online study employed a 2 × 2 between‐subjects design, manipulating group membership (ingroup vs. outgroup) and valence (positive vs. negative) of interactions. After obtaining informed consent, participants were randomly assigned to one of four conditions: a positive or negative interaction with an outgroup member, a Muslim high school‐aged girl (Sana) and a positive or negative interaction with an ingroup member (non‐Muslim high school‐aged Italian girls). These conditions were depicted through video manipulations sourced from the TV series ‘Skam Italia’. This approach allowed us to construct ecologically valid and condition‐specific vicarious contact stimuli, while holding the viewing context and approximate duration (around 5 min) constant across conditions. Excerpts were selected to include a brief contextual introduction followed by focal interaction segments matching the assigned condition: positive or negative interactions involving either Sana as the Muslim outgroup member or non‐Muslim Italian ingroup members. We used excerpts from a mainstream television series because vicarious intergroup contact frequently occurs through mass media exposure, making such stimuli ecologically relevant and a realistic channel for mediated intergroup contact experiences (White et al., 2021). We assessed participants' perceptions of the clips (e.g. plausibility/realism and emotional tone) via manipulation checks. Utilizing clips from a TV series was permissible under copyright law (art. 70 L. 633/1941), allowing partial reproduction for non‐commercial research purposes.

The videos facilitated vicarious intergroup contact by depicting interactions between ingroup (non‐Muslim Italian girls) and outgroup (Muslim girl) members. Each video started with an introduction to the characters and the context, followed by either positive or negative interactions depending on the assigned condition. In the *outgroup positive* condition, Italian girls engaged in positive interactions (e.g. comforting, reassurance) with an outgroup member (Muslim girl), while the *outgroup negative* condition involved negative interactions (e.g. sharp arguments, visible distress) with the same character. In contrast, in the *ingroup positive* condition, Italian girls had positive interactions among ingroup members (other Italian girls), while the *ingroup negative* condition featured negative interactions among ingroup members. A full description of the content of the videos is reported in the Supplementary Materials. Following the video viewing, participants completed the scales reported below, with a break between cognitive liberalization outcomes and moderators.

#### Measures

##### Abstract reasoning

Six items of varying complexity—after two practice items—from the Matrix Reasoning Item Bank (MaRs‐IB; Chierchia et al., 2019) were employed. Participants had to select a figure that would complete a 9‐figure matrix within 30 s. The abstract reasoning (MARS) score was quantified by the total number of correct answers from 0 to 6.

##### Creative convergent thinking

Creative convergent thinking was assessed using the Remote Associates Test (RAT; Mednick, 1962; Italian validation: Salvi et al., 2020). Participants were presented with three seemingly unrelated words and were asked to find—in 30 s maximum—a fourth word that could be linked to the original three through one of three associations: synonymy, semantic association or forming a compound word. We employed six three‐word problems; however, as in Study 1, we miswrote one of the three words in a problem, we used only five.

##### Creativity

We employed the Alternative Uses Task (AUT; Guilford, 1967): participants were given a common, everyday object, in our case a pencil, and were asked to generate as many alternative uses for the object as possible within a 2‐min time limit. Responses were scored across four dimensions: fluency (the total number of uses generated), elaboration (the level of detail in all responses), flexibility (the variety of different categories the responses covered) and originality (the rareness of the responses). Rules adopted to code categories are reported in the Supplementary Materials.

##### Self‐report cognitive flexibility

Cognitive flexibility was measured using four items (‘I can communicate an idea in many different ways’, ‘I avoid new and unusual situations’, ‘I have many possible ways of behaving in a given situation’ and ‘I am willing to listen and consider alternatives for handling a problem’) adapted from the Cognitive Flexibility Scale (Martin & Rubin, 1995; 7‐point response scale, from 1 = *strongly disagree* to 7 = *strongly agree*). Given the very low internal consistency of the scale (α = .26), items were analysed individually rather than as a composite measure.

##### Cultural deprovincialization

We employed four items (‘Getting to know individuals from different cultures makes me feel more open towards other people’, ‘Knowing customs and traditions of different cultures helps me feel closer to other people’, ‘Noticing cultural differences makes me feel less open and less friendly to other people’—reverse—‘I am willing to expand my circle of friends to people from different cultures’) adapted from the Cultural Deprovincialization Scale (CDS; Boin et al., 2020; α = .69; 7‐point response scale from 1 = *does not describe me at all* to 7 = *describes me very well*), which assesses the outgroup‐oriented facet of deprovincialization, namely a curious and open stance towards other cultures, customs and worldviews.

##### Group deprovincialization

We employed four items (‘One should try to adopt a broader cultural perspective than only the perspective of one's own culture’, ‘How we perceive the world in our country is just one of many possibilities’, ‘One should nuance one's own cultural worldview and not make it sacred’ and ‘One has to learn to look beyond the limits of one's own national culture’) adapted from the Group Deprovincialization Scale (GDS; Martinovic & Verkuyten, 2013; α = .75; 7‐point response scale from 1 = *completely disagree* to 7 = *completely agree*), which measures the ingroup‐oriented facet of deprovincialization, namely the belief that the ingroup's worldview is not the only legitimate one.

##### Environmental concern

We used four items adapted from those used by Meleady et al. (2020): ‘If things continue on their present course, we will soon experience serious environmental problems’; ‘People have been giving far too little attention to how human progress has damaged the environment’; ‘Climate change will not affect life on Earth in any significant way’; and ‘Climate change is natural and not due to human influence’, with the last two reverse‐coded (response scale from 1 = *completely disagree* to 7 = *completely agree*), with higher scores indicating greater concern for the environment (α = .64).

##### Curiosity

The Joyous Exploration dimension of the Five‐Dimensional Curiosity Scale (Kashdan et al., 2018) was used to measure participants' curiosity about new experiences and worldviews (α = .73). The scale consisted of six items (example item: ‘I always seek experiences that challenge my conceptions of myself and the world’; response scale from 1 = *does not describe me at all* to 7 = *describes me very well*).

##### Social dominance orientation

We employed the 8‐item SDO‐Dominance (SDO‐D) subscale of the SDO7 scale (Ho et al., 2015; example item: ‘Some groups of people are simply inferior to other groups’; α = .79; response scale from 1 = *completely disagree* to 7 = *completely agree*).

##### Need for cognitive closure

We used the 15‐item Need for Cognitive Closure Scale (NFC; Roets & Van Hiel, 2011; example item: ‘I don't like to go into a situation without knowing what I can expect from it’; α = .80; response scale from 1 = *completely disagree* to 7 = *completely agree*), which measures desire for definitive answers and aversion to ambiguity.

##### Intergroup contact

Because previous contact experiences with Muslim people may influence intergroup outcomes, we assessed positive and negative direct contact as theoretically relevant control variables and included them as covariates in robustness analyses in line with the pre‐registration. Contact was measured with two items for both positive and negative experiences (capturing quantity and frequency), adapted from Boin et al. (2024): ‘How many Muslim people do you have mostly positive [negative] interactions with?’ (response scale: 1 = none; 2 = 1–2; 3 = 3–4; 4 = 5–6; 5 = more than 7) and ‘How often do you deal with Muslim people and experience it as a positive [negative]?’ (response scale: 1 = never, 2 = rarely, 3 = sometimes, 4 = often, 5 = very often). The two items were averaged separately for positive (ρ = .78) and negative (ρ = .72) contact.

##### Manipulation check questions

Besides the abovementioned attention checks, we had a series of additional check questions to test whether the manipulations produced the expected psychological effects. We asked participants (response scale from 1 = *not at all* to 7 = *very much*) about the emotions they felt watching the video (‘How much did you experience positive emotions while watching the video?’; ‘How much did you experience negative emotions while watching the video’), the plausibility of the video (‘To what extent did the situations represented in the video seem plausible to you, that is, they could happen in everyday life?’), difficulty in understanding the videos (‘How difficult was it to understand the situations you saw portrayed in the video?’) and perceived valence of the video (‘To what extent did the situations represented in the video seem positive [negative] to you?’).

### Results

Table S1 of the Supplementary Material reports means, standard deviations and intercorrelations of study measures.

#### Manipulation checks

Two‐way ANOVAs confirmed that the valence manipulation was effective: positive‐contact videos were perceived as more positive and less negative and elicited more positive and fewer negative emotions. Some smaller differences also emerged, with outgroup videos being perceived as somewhat more positive, plausible and easier to understand than ingroup videos. Full results are reported in the Supplementary Materials.

#### Testing the hypotheses

To address Aim 1, we tested H1 by performing linear regression models in which the dependent variables representing cognitive liberalization were predicted by the two factors, that is, *Valence* and *Group Membership*, as well as their interaction.^2^ Although this slightly deviated from the pre‐registered plan, which specified three dummy‐coded variables with one reference category, this approach was adopted because it more directly reflects the 2 × 2 factorial design. Predictors were entered as zero–one dummy‐coded variables. As shown in Table 1, we only found a statistically significant effect of valence on the flexibility index of creativity (full results are reported in Tables S3–S6).

**TABLE 1:** Study 1: the effects of the manipulations (Group Membership and Valence) on the flexibility creativity index.

	Flexibility—AUT				
	*b*	*SE*	β	95% CI	*p*
Intercept	4.60	0.25	.00	4.11, 5.09	<.001
Outgroup	0.09	0.36	−.08	−0.62, 0.80	.801
Positive	0.94	0.37	.08	0.22, 1.66	.011
Outgroup × Positive	−1.01	0.52	−.10	−2.02, 0.01	.052
*R* ^2^	0.022				

*Note*: AUT = Alternative Uses Task (creativity). Outgroup (0 = ingroup interaction, 1 = outgroup interaction) = effect of watching the video including an interaction with the outgroup compared to an interaction with the ingroup (Group Membership factor); Positive (0 = negative interaction, 1 = positive interaction) = effect of watching the video including positive contact compared to negative (Valence factor).

**FIGURE 1:**
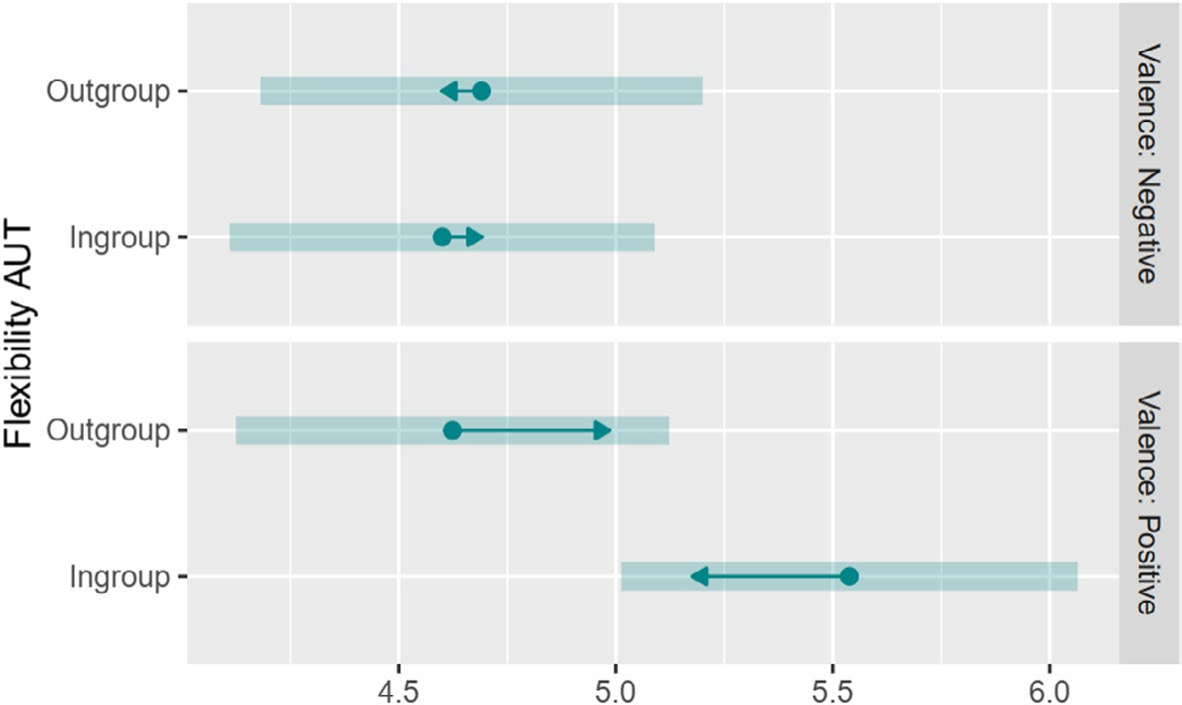
Study 1: comparison of estimated marginal means, model for flexibility Alternative Uses Task (AUT) (Table 1). Estimated marginal means for the flexibility index of the AUT from the model testing the Valence × Group Membership effect. Error bars represent 95% confidence intervals. The pattern indicates higher flexibility after positive ingroup contact than after positive outgroup contact, whereas the negative‐contact conditions did not differ.

Results indicated that watching a positive interaction, as opposed to a negative one, increased the flexibility index of our creativity measure. Furthermore, the interaction between the two conditions was not statistically significant (*p* = .052). Results of Tukey‐adjusted pairwise comparisons of estimated marginal means are shown in Figure 1 (points are estimated marginal means; bars are their 95% CI; statistically significant differences are given by non‐overlapping arrows) and revealed no statistically significant difference (*t* (390) = −0.252, *p* = .801, *d* = 0.35) between the *ingroup negative* (*M* = 4.60, *SD* = 0.25) and *outgroup negative* (*M* = 4.69, *SD* = 0.26) conditions. However, the flexibility dimension of creativity was higher (*t* (390) = 2.48, *p* = .014, *d* = 3.54) in the *ingroup positive* (*M* = 5.54, *SD* = 0.27) compared to the *outgroup positive* (*M* = 4.62, *SD* = 0.25) condition, contrary to H1 and the cognitive liberalization hypothesis.

To address Aim 3, we tested H3 by extending the models performed to test H1 to include the dispositions of SDO, NFC, and dispositional curiosity, along with their interactions with the experimental conditions (full details can be found in Tables S7–S15). To maintain model parsimony and minimize collinearity, each disposition and its interactions were added separately. No statistically significant interaction terms were found for curiosity. However, we found a statistically significant three‐way interaction between SDO and the experimental conditions for abstract reasoning () and two statistically significant three‐way interactions between NFC and the experimental conditions for fluency and elaboration indexes of creativity (see Table 2).

**TABLE 2:** Study 1: the moderation of SDO in the model with MARS, and of NFC in the model with fluency and elaboration creativity indices.

	MARS					Fluency—AUT					Elaboration—AUT				
	*b*	*SE*	β	95% CI	*p*	*b*	*SE*	β	95% CI	*p*	*b*	*SE*	β	95% CI	*p*
Intercept	3.42	0.27	.01	2.90, 3.95	<.001	5.60	1.94	−.00	1.79, 9.41	.004	7.67	2.52	−.00	2.71, 12.63	.003
Outgroup	0.26	0.40	−.05	−0.52, 1.04	.510	5.43	2.80	−.05	−0.07, 10.93	.053	5.57	3.64	−.04	−1.59, 12.72	.127
Positive	0.47	0.43	−.02	−0.37, 1.31	.269	7.72	2.74	.03	2.34, 13.11	.005	8.16	3.56	.04	1.16, 15.17	.023
Outgroup × Positive	−1.44	0.59	−.06	−2.61, −0.27	.016	−9.48	4.00	−.06	−17.35, −1.62	.018	−11.22	5.21	−.03	−21.46, −0.98	.032
SDO	−0.05	0.12	−.05	−0.28, 0.17	.646										
Outgroup × SDO	−0.12	0.17	.06	−0.46, 0.21	.471										
Positive × SDO	−0.19	0.20	.04	−0.58, 0.20	.348										
Outgroup × Positive × SDO	0.59	0.28	.11	0.04, 1.14	.036										
NFC						0.47	0.44	−.10	−0.41, 1.34	.295	0.31	0.58	−.10	−0.82, 1.45	.588
Outgroup × NFC						−1.27	0.64	−.03	−2.53, −0.01	.049	−1.34	0.84	−.01	−2.98, 0.30	.109
Positive × NFC						−1.65	0.64	−.07	−2.91, −0.40	.010	−1.76	0.83	−.04	−3.39, −0.12	.035
Outgroup × Positive × NFC						2.01	0.93	.11	0.18, 3.84	.031	2.49	1.21	.10	0.11, 4.87	.040
*R* ^2^	0.025					0.038					0.029				

*Note*: Outgroup (0 = ingroup interaction, 1 = outgroup interaction) = effect of (see suggestions above) interaction with the outgroup compared to the interaction with the ingroup (Group Membership factor); Positive (0 = negative interaction, 1 = positive interaction) = effect of positive intergroup contact compared to negative (Valence factor).

Abbreviations: AUT, Alternative Uses Task (creativity); NFC, Need for Closure; SDO, Social Dominance Orientation.

**FIGURE 2:**
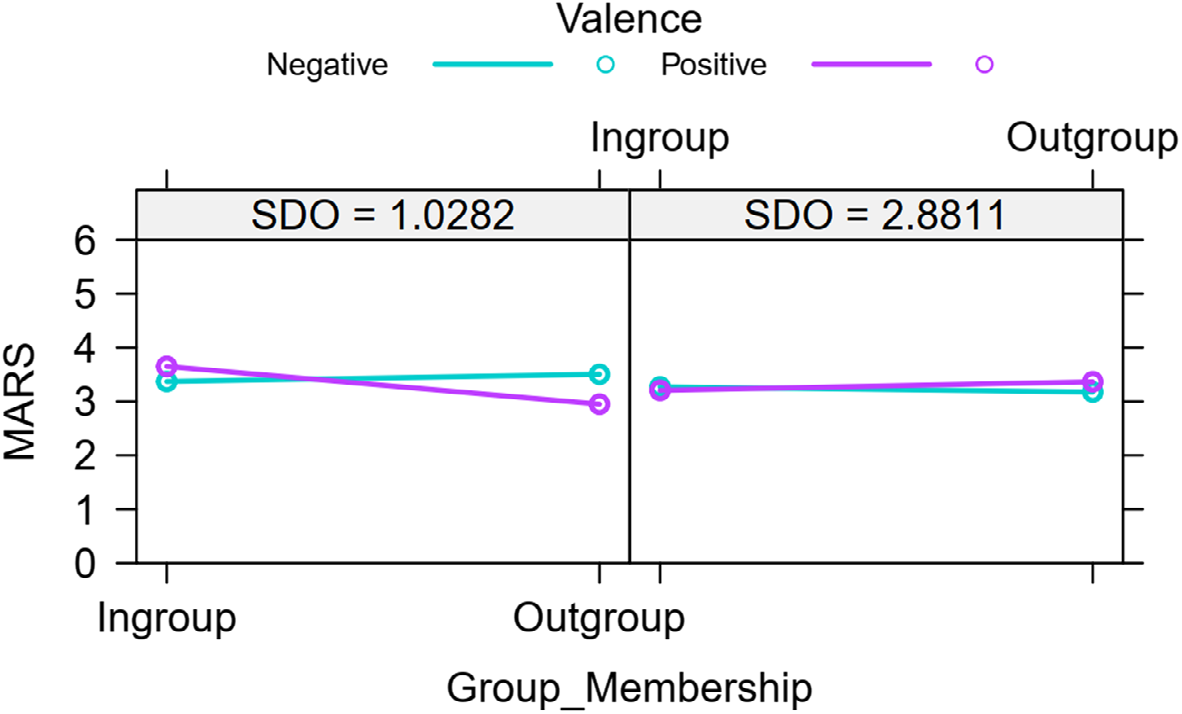
Study 1: the interaction among experimental conditions and social dominance orientation (SDO), model for abstract reasoning score (MARS). Predicted MARS scores from the model testing the three‐way interaction between SDO, interaction valence and group membership. Simple‐slope analyses showed no effect of condition at high levels of SDO. At low SDO levels, positive outgroup contact predicted lower MARS scores than both negative outgroup contact (β = −.23, *b* = −0.55, *SE* = 0.25, *p* = .025) and positive ingroup contact (β = −.28, *b* = −.70, *SE* = 0.25, *p* = .006).

Decomposing the three‐way interaction between the conditions and SDO (Figure 2) through simple slopes, we found that when SDO was high (+1 SD, SDO = 2.88), the manipulations were not associated with MARS. When SDO was low (−1 SD, SDO = 1.02), being exposed to a positive compared to a negative interaction with the outgroup was associated with lower MARS scores (β = −.23, *b* = −0.55, *SE* = 0.25, *p* = .025). When SDO was low, being exposed to a positive interaction with the outgroup compared to a positive interaction with the ingroup was also associated with lower MARS scores (β = −.28, *b* = −0.70, *SE* = 0.25, *p* = .006).

**FIGURE 3:**
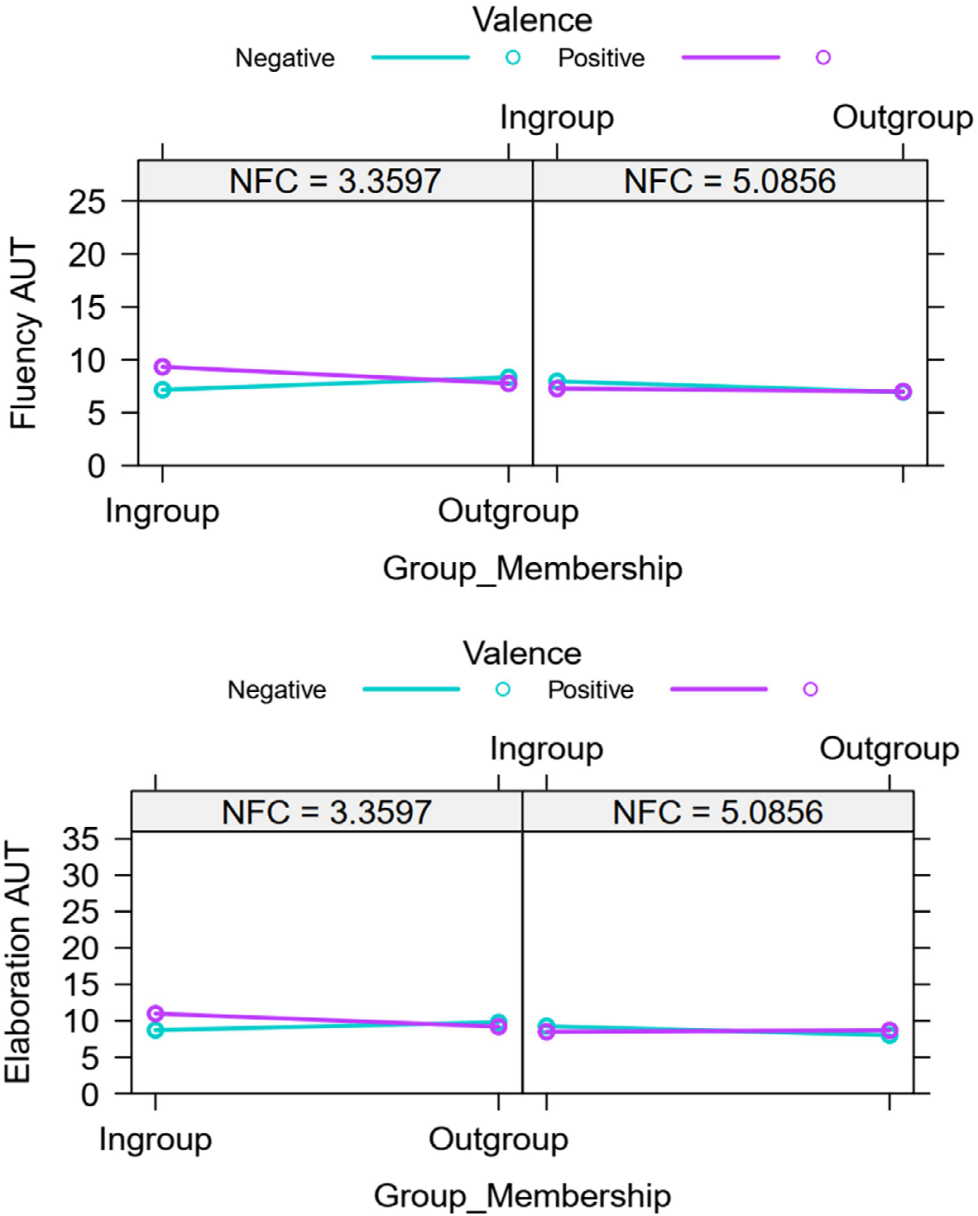
Study 1: the interaction among experimental conditions and need for closure (NFC), models for fluency and elaboration (Alternative Uses Task, AUT). Predicted fluency and elaboration scores from the AUT models testing the three‐way interaction between need for cognitive closure, interaction valence and group membership. Simple‐slope analyses indicated that condition was not associated with fluency or elaboration at high NFC. At low NFC, positive ingroup contact predicted higher fluency (β = .27, *b* = 2.17, *SE* = 0.78, *p* = .006) and elaboration (β = .22, *b* = 2.26, *SE* = 1.02, *p* = .026) than negative ingroup contact; for fluency, positive outgroup contact predicted lower scores than positive ingroup contact (β = −.19, *b* = −1.56, *SE* = 0.79, *p* = .049).

We then decomposed the three‐way interactions between NFC and the conditions in the models with fluency and elaboration index of creativity as the dependent variables (see Figure 3): when NFC was high (+1 SD, NFC = 5.09), conditions were not associated with fluency and elaboration indexes of AUT. When NFC was low (−1 SD, NFC = 3.36), watching a positive—compared to a negative—interaction with the ingroup was associated with higher fluency (β = .27, *b* = 2.17, *SE* = 0.78, *p* = .006) and elaboration (β = .22, *b* = 2.26, *SE* = 1.02, *p* = .026). Moreover, for fluency, when NFC was low, being exposed to a positive intergroup interaction compared to a positive ingroup interaction was associated with lower fluency scores (β = − .19, *b* = −1.56, *SE* = 0.79, *p* = .049).

## STUDY 2

### Method

The Psychological Research Ethics Committee of the University of Padova approved the procedures of this study, protocol #5260. Data, R script, video description and pre‐registration are available on OSF (https://doi.org/10.17605/OSF.IO/34H2E).

#### Participants

Participants were recruited in the same way as Study 1. As in Study 1, employing the same settings, a priori power analysis gave us the minimum sample size of 351 participants. The questionnaire was completed by 722 participants. Using the criteria of Study 1, we excluded participants who self‐identified as Muslim (*N* = 5), did not provide consent (*N* = 9), made more than one error in attention manipulation checks (*N* = 37), spent more than 400 s (video duration: around 271 s) on the video page (*N* = 53) and took too much (more than 3600 s, i.e. 1 h) or too little (<1000 s) time to complete the questionnaire (*N* = 44). After applying these exclusions, the final sample size was *N* = 574^2^. A sensitivity analysis (α = .05, 1 − β = .80) showed that the design had adequate power to detect effects of *f* = 0.12 (*f* ^*2*^ = 0.01, *R* ^*2*^ = 0.01), that is, in the small‐to‐medium range.

Participants' age ranged between 18 and 77 years (*M* = 29.86, *SD* = 14.87). Regarding gender, 339 (59%) participants declared themselves as women, 230 (40%) as men and 5 (1%) as non‐binary. Their education levels were varied: the highest education level attained was primary school for 0.2%, secondary school for 7.9%, a high school diploma for 71.1%, a bachelor's degree for 9.8% and higher degrees (master's or PhD) for 10.8% of the sample.

#### Procedure

The procedure was similar to Study 1. The study employed a 2 × 2 between‐subject design, manipulating *Semantic distance* (atypical vs. prototypical outgroup member) and *Valence* (positive vs. negative) in intergroup interactions, through 5‐min video manipulations with intergroup contact scenes taken from the TV series ‘Skam Italia’, thereby creating vicarious contact for viewers. As in Study 1, excerpts were selected to include a brief contextual introduction followed by focal interaction segments matching the assigned condition: positive or negative interactions with Sana portrayed through prototypical or atypical behaviours in relation to Muslim identity in the Italian context. After obtaining informed consent, participants were randomly assigned to one of four conditions: a positive or negative interaction with a prototypical outgroup member or a positive or negative interaction with an atypical outgroup member. All videos had an introductory part to present characters and context, then they varied according to the condition. In the prototypical conditions, the outgroup member enacted behaviours commonly associated with a prototypical Muslim identity in the Italian context (e.g. visible religious practice, abstaining from alcohol, fasting, discomfort with explicit sexual talk). In the atypical conditions, she enacted behaviours less typical of that identity (e.g. driving herself, watching a men's basketball game, dancing and toasting at a club). Within each condition, the interaction with non‐Muslim peers was either positive or negative, yielding four conditions. A full description of the video content is provided in the Supplementary Materials.

#### Measures

In Study 2, we used the same measures for the dependent variables that we used in Study 1: abstract reasoning (MARS), convergent thinking measured (RAT), self‐reported cognitive flexibility (with items treated separately because α = .35), creativity, group deprovincialization (α = .80), cultural deprovincialization (α = .67), environmental concern (α = .59), curiosity (α = .75), SDO (α = .81), NFC (α = .79) and intergroup contact (positive: ρ = .74; negative: ρ = .79). As in Study 1, we asked participants additional manipulation check questions. Such questions were ‘To what extent were you aware of the fact that you and Sana are part of different groups?’ ‘To what extent did you perceive Sana as typical and representative of Muslim people in general?’ (response scale: from 1 = *not at all* to 7 = *very much*). Then, we employed the questions of Study 1 asking participants about their positive and negative emotions felt during the video, the plausibility of the portrayed situations, difficulty to understand the video and perceived positive and negative valence of the video. As in Study 1, we also asked participants if they have ever watched Skam Italia, and which season(s).

### Results

Table S16 of the Supplementary Material reports means, standard deviations and intercorrelations of study measures.

#### Manipulation checks

Manipulation checks confirmed the valence manipulation and showed that ‘semantic distance’ affected perceived prototypicality (which was also modestly higher under negative vs. positive valence). Additional differences across conditions also emerged (e.g. atypical videos were perceived as somewhat more difficult to understand and elicited slightly less negative emotional reactions). Full manipulation‐check results are reported in the Supplementary Materials.

#### Testing the hypotheses

Following Aim 2, we tested H2 by computing linear regression models in which the dependent variables portraying cognitive liberalization were predicted by the two factors, that is, *Semantic distance* and *Valence*, and their interaction^2^. We found statistically significant effects of the manipulations in three models (see Table 3): for the dependent variables cultural deprovincialization, group deprovincialization and the flexibility index of creativity (other results are fully reported in the Tables S18–S20).

**TABLE 3:** Study 2: the effects of the manipulations (semantic distance and valence) on cognitive liberalization variables.

	Cultural deprovincialization					Group deprovincialization					Flexibility—AUT				
	*b*	*SE*	β	95% CI	*p*	*b*	*SE*	β	95% CI	*p*	*b*	*SE*	β	95% CI	*p*
Intercept	6.01	0.08	.00	5.85, 6.17	<.001	6.36	0.07	−.00	6.21, 6.51	<.001	5.44	0.22	.00	5.00, 5.88	<.001
Atypical	−0.28	0.11	−.06	−0.50, −0.06	.015	−0.21	0.10	−.13	−0.41, −0.00	.045	−0.61	0.31	−.09	−1.22, −0.01	.046
Positive	−0.14	0.11	.02	−0.36, 0.09	.236	0.00	0.10	−.01	−0.20, 0.20	.984	−0.39	0.31	−.04	−1.00, 0.21	.202
Atypical × Positive	0.34	0.16	.09	0.03, 0.66	.033	−0.03	0.14	−.01	−0.31, 0.26	.858	0.34	0.43	.03	−0.51, 1.19	.434
*R* ^2^	0.011					0.016					0.010				

*Note*: Atypical (0 = typical interaction, 1 = atypical interaction) = effect of an atypical outgroup member compared to a prototypical one (Semantic distance factor); Positive (0 = negative interaction, 1 = positive interaction) = effect of positive intergroup contact compared to negative (valence factor).

Abbreviations: AUT, Alternative Uses Task (creativity).

**FIGURE 4:**
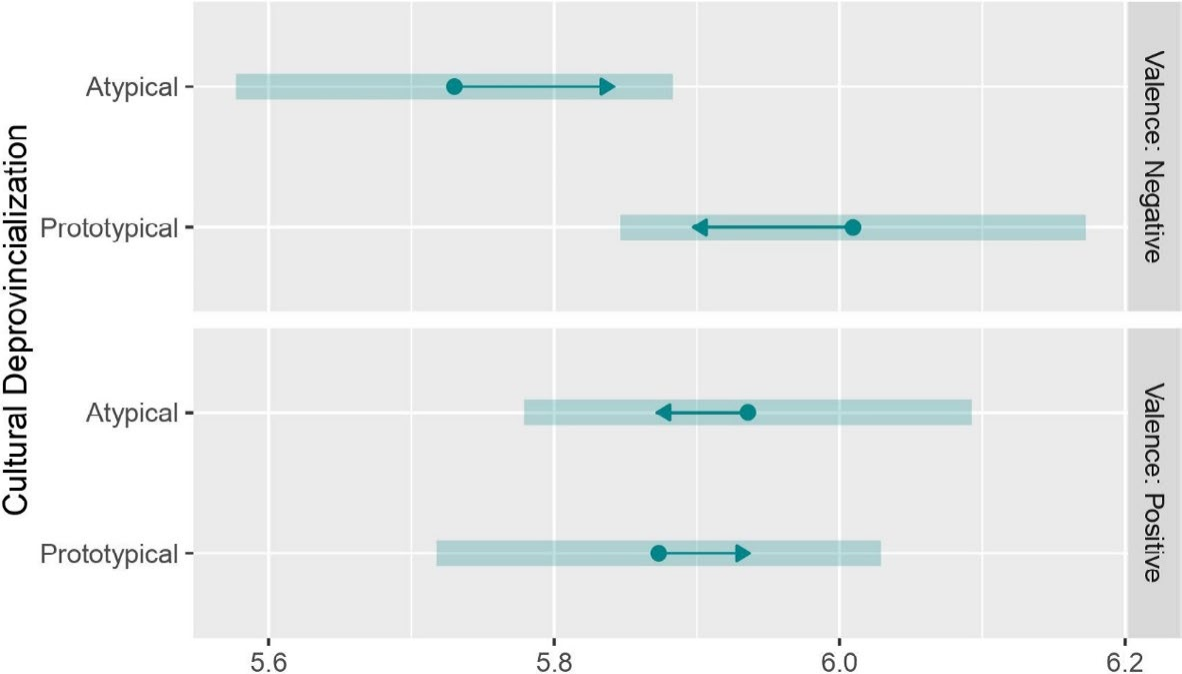
Study 2: comparison of estimated marginal means, model for cultural deprovincialization (Table 3). Estimated marginal means for cultural deprovincialization from the model testing the Semantic Distance × Valence effect. Error bars represent 95% confidence intervals. The pattern indicates lower cultural deprovincialization after atypical‐negative than after prototypical‐negative contact, whereas the positive‐contact conditions did not differ.

Results showed that being exposed to vicarious contact with an atypical—compared to a prototypical—outgroup member decreased cultural deprovincialization, group deprovincialization and the flexibility dimension of creativity; this effect is contrary to both H2 and the cognitive liberalization hypothesis, regarding the role of semantic distance. Moreover, we found a statistically significant interaction of the two conditions in the model for cultural deprovincialization. Tukey‐adjusted pairwise comparisons of estimated marginal means (plotted in Figure 4) showed that while the *prototypical positive* (*M* = 5.87, *SD* = 0.96) and *atypical positive* (*M* = 5.94, *SD* = 0.89) videos did not show any difference (*t* (570) = −0.555, *p* = .579, *d* = 0.08), cultural deprovincialization was lower in the *atypical negative* (*M* = 5.73, *SD* = 1.07) compared to the *prototypical negative* (*M* = 6.01, *SD* = 0.90) condition (*t* (570) = 2.45, *p* = .015, *d* = 0.28).

Following Aim 3, we tested H3 by adding to all the models testing H2 SDO, NFC and curiosity and their interactions with the experimental conditions (full results in Tables S21–S29). To avoid collinearity and keep model parsimony, we included each disposition separately. We did not find any statistically significant interaction term with NFC and curiosity; regarding models with SDO, we found one statistically significant interaction when the dependent variable was cultural deprovincialization (Table 4).

**TABLE 4:** Study 2: the moderation of SDO in the model for cultural deprovincialization.

	Cultural deprovincialization				
	*b*	*SE*	β	95% CI	*p*
Intercept	6.71	0.18	.00	6.36, 7.06	<.001
Atypical	0.12	0.25	−.01	−0.36, 0.60	.628
Positive	0.20	0.25	.01	−0.29, 0.68	.425
SDO	−0.37	0.09	−.40	−0.54, −0.20	<.001
Atypical × Positive	−0.65	0.34	.06	−1.33, 0.02	.058
Atypical × SDO	−0.13	0.11	.05	−0.34, 0.09	.248
Positive × SDO	−0.15	0.12	.04	−0.37, 0.08	.201
Atypical × Positive × SDO	0.44	0.15	.11	0.13, 0.74	.005
*R* ^2^	0.191				

*Note*: atypical (0 = typical interaction, 1 = atypical interaction) = effect of an atypical outgroup member compared to a prototypical (semantic distance factor); positive (0 = negative interaction, 1 = positive interaction) = effect of positive intergroup contact compared to negative (valence factor).

Abbreviations: SDO, social dominance orientation.

**FIGURE 5:**
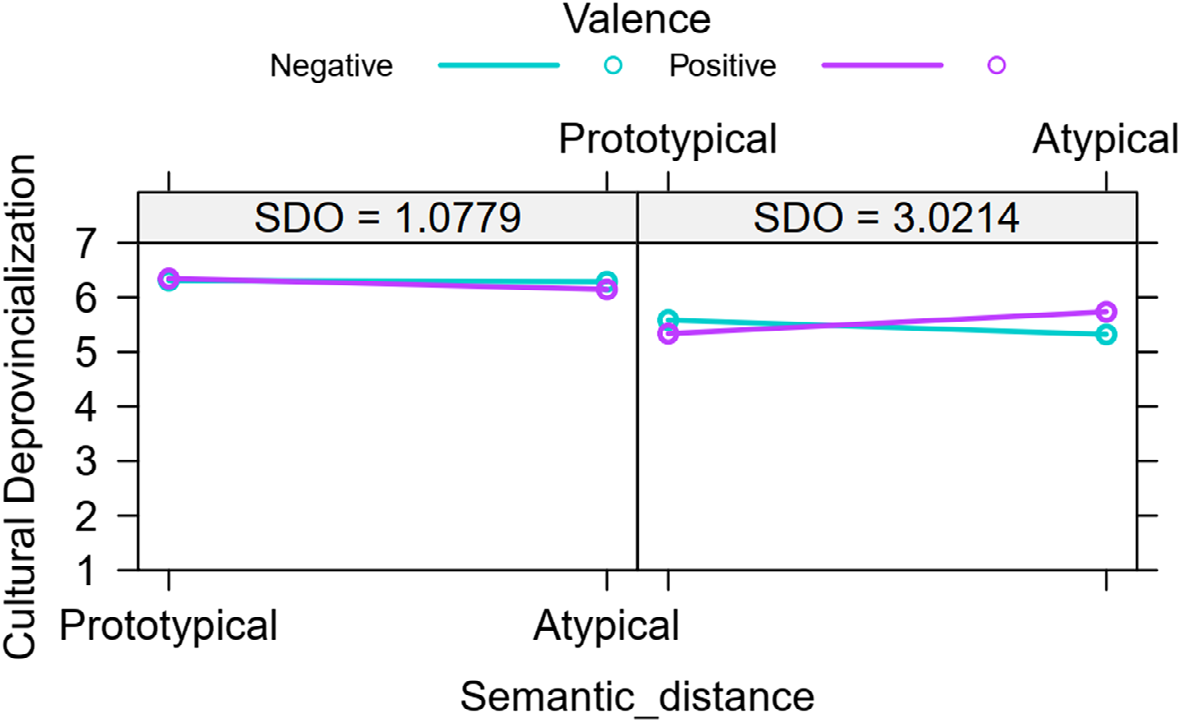
Study 2: the interaction among experimental conditions and social dominance orientation (SDO), model for cultural deprovincialization. Predicted cultural deprovincialization from the model testing the three‐way interaction between SDO, semantic distance and interaction valence. Simple‐slope analyses indicated no effects at low SDO. At high SDO, atypical‐positive contact predicted higher cultural deprovincialization (β = .21, *b* = 0.40, *SE* = 0.15, *p* = .007) than both prototypical‐positive and atypical‐negative contact (β = .23, *b* = 0.41, *SE* = 0.14, *p* = .002).

We decomposed the three‐way interaction between the conditions and SDO (Figure 5) through simple slopes: when SDO was low (−1 SD, SDO = 1.08), the manipulations had no effects. When SDO was high (+1 SD, SDO = 3.02), being exposed to a positive interaction with an atypical—compared to a prototypical—outgroup member was positively associated with higher cultural deprovincialization (β = .21, *b* = 0.40, *SE* = 0.15, *p* = .007; purple line in the graph). When SDO was high, vicarious negative contact with an atypical—compared to a prototypical—outgroup member was not associated with deprovincialization, although with a tendency for a negative association (β = −.13, *b* = −0.26, *SE* = 0.15, *p* = .083; turquoise line). When SDO was high, being exposed to an atypical positive compared to an atypical negative outgroup member was associated with higher deprovincialization (β = .23, *b* = 0.41, *SE* = 0.14, *p* = .002).

## DISCUSSION

Across two sufficiently powered and pre‐registered studies, we examined the TTE of vicarious intergroup contact on several indices of cognitive liberalization. Because we included both performance‐based cognitive indicators (MARS, RAT and AUT), self‐reported cognitive flexibility and self‐report socio‐cultural indicators (cultural and group deprovincialization), we were able to provide a multi‐method test of TTEs. Accordingly, we interpret effects that converge across measurement types as particularly robust, while also recognizing that performance‐based and self‐report indicators may capture partly distinct components of cognitive liberalization and therefore need not show identical patterns following a brief exposure. Contrary to our expectations, the findings provided limited support for the TTE hypothesis. In Study 1, positive vicarious interactions enhanced creativity, but mainly in the *ingroup*‐positive condition, challenging the predicted superiority of positive *intergroup* interactions. In Study 2, exposure to interactions with atypical outgroup members unexpectedly reduced cultural deprovincialization, group deprovincialization and creativity.

An important consideration concerns the performance‐based cognitive liberalization measures [i.e. abstract reasoning (MARS) and creative convergent thinking (RAT) and the AUT indices], which may primarily reflect stable individual differences rather than state‐like fluctuations. This constraint implies that any effects of a single, brief vicarious contact exposure are expected to be small and therefore difficult to detect, even if contact is meaningfully related to these outcomes at the between‐person level. This interpretation aligns with recent longitudinal evidence showing robust between‐person associations between intergroup contact and cognitive‐related outcomes, but limited evidence for within‐person changes following short‐term increases in contact, and for shifts in cognitive skills over short intervals (Friehs et al., 2025; Shulman et al., 2025). An additional consideration concerns the manipulation checks. Although the intended manipulations were successful, some smaller unintended differences between conditions also emerged and may have influenced engagement with the stimuli. In Study 1, outgroup videos were perceived as slightly more positive, plausible and easier to understand than ingroup videos, whereas in Study 2 atypical videos were somewhat harder to understand and elicited slightly less negative emotional responses. Although modest in magnitude these differences may have partly contributed to the limited evidence for TTE effects.

Interestingly, we found interaction effects suggesting moderation of individual differences. First, in Study 1, low‐SDO participants experienced reduced abstract reasoning following positive intergroup interactions, and low‐NFC respondents showed reduced fluency and elaboration index of creativity following negative ingroup interactions. All three interaction effects go against the TTE (as negative vicarious *inter*group contact was related to *lower* cognitive functioning, or the effects were found merely when watching *in*group contact experiences). This also goes against the ‘prone‐to‐prejudice’ hypothesis of Turner et al. (2020), which stated that contact effects would be as pronounced, if not more pronounced, among people that are high in SDO or NFC. Here, 11 out of 14 interaction effects point to equally weak effects of our manipulations for those keen vs. unkeen to prejudice, and the 3 statistically significant ones indicate that those least keen to prejudice seem to be impacted to a larger extent. This pattern could also be due to the dependent variables not being measures of prejudice, and none of them—except the two deprovincialization scales—reflecting an intergroup dynamic.

Second, in Study 2, there was one notable interaction effect: participants high in SDO showed higher cultural deprovincialization following positive interactions with atypical outgroup members, suggesting evidence for a TTE among those most ‘prone‐to‐prejudice’ in this socio‐cultural dimension of liberalization. Here too, most results lead us to reject the TTE, but one promising finding was uncovered by adding the nuanced individual differences approach.

### Theoretical and practical implications

Theoretically, the findings suggest that the cognitive liberalization hypothesis may not generalize across all intergroup contexts. Instead, the benefits of vicarious contact may depend on the nature of the contact situation, group prototypicality, valence and individual characteristics (e.g. SDO, NFC). This highlights the importance of further refining models of intergroup contact to account for differential effects based on individual and situational factors.

First, the power of *indirect* intergroup experiences for TTE should be further explored, including extended or online contact. Second, cognitive liberalization theory predicted that atypical outgroup members could challenge existing stereotypes and stimulate cognitive flexibility (Meleady et al., 2019); however, our findings showed that contact with atypical members reduced deprovincialization and creativity. Importantly, the manipulation checks indicate that the semantic‐distance manipulation operated on the intended dimension: participants perceived Sana as less typical in the atypical than in the prototypical conditions, whereas their awareness of belonging to a different group than Sana did not differ across conditions. This specificity is a strength of the manipulation because it suggests that the videos varied perceived behavioural prototypicality without confounding this variation with differences in intergroup salience. At the same time, this pattern is theoretically informative. The atypical videos may have portrayed Sana as behaviourally non‐prototypical while still keeping her Muslim identity salient. Rather than producing individualized processing, identity‐salient atypicality may have increased the complexity of the interaction, potentially explaining why atypicality did not yield the expected cognitive liberalization effects. Relatedly, atypicality may have imposed greater cognitive demands on participants, especially in vicarious contact scenarios where the observer lacks direct engagement (Fiske & Neuberg, 1990). This cognitive load could inadvertently hinder creative and open‐minded thinking. Future research should consider how the complexity and effort required to process atypical exemplars interact with the specific cognitive outcomes of interest.

Third, the valence of intergroup contact affects not only attitudes but also cognitive outcomes (Pettigrew & Tropp, 2008). Positive interactions are hypothesized to foster openness and creativity by eliciting favourable emotions and reducing anxiety (Paolini et al., 2010). The present study found that positive valence increased creativity but primarily in ingroup‐positive conditions, suggesting that observers may derive greater psychological comfort and emotional resonance from observing harmonious ingroup dynamics. Negative interactions, conversely, can amplify intergroup anxiety and stereotype salience (Paolini et al., 2010), potentially undermining cognitive liberalization. However, the nuanced role of negativity in vicarious contexts remains underexplored. For example, interactions that are constructively critical or involve resolution of intergroup conflict might yield different cognitive benefits compared to uniformly negative interactions.

Fourth, high‐SDO individuals, typically characterized by hierarchical worldviews and resistance to equality (Sidanius & Pratto, 1999), exhibited greater cultural deprovincialization when exposed to positive interactions with atypical outgroup members. This finding aligns with prior work suggesting that high‐SDO individuals may benefit disproportionately from well‐structured intergroup contact (Hodson, 2011). Similarly, low‐NFC participants showed higher creativity, but only in positive ingroup conditions. These patterns underscore the importance of tailoring intergroup contact interventions to individual cognitive styles. For example, high‐NFC individuals might benefit more from structured and predictable contact scenarios, whereas low‐NFC individuals may thrive in unstructured or novel contexts.

Practically, these results caution against one‐size‐fits‐all interventions for promoting cognitive liberalization through intergroup contact. Efforts to enhance open‐mindedness and creativity via media‐based or vicarious intergroup exposure must consider the composition of target audiences, the nature of portrayed interactions and the balance between cognitive challenge and emotional accessibility. For instance, alternating portrayals of prototypical and atypical outgroup members may stimulate diverse cognitive processes.

### Limitations and future directions

This study has some limitations. First, the present studies provide a controlled test of a single, brief, standardized vicarious exposure. This design compared condition‐specific effects of valence, group membership and prototypicality using ecologically meaningful media stimuli, that is, videos from a real and popular TV series. At the same time, cognitive liberalization may be facilitated by cumulative exposure to outgroup perspectives, stronger narrative engagement or more reciprocal forms of interaction. Future studies could employ repeated exposures, for example, by comparing full‐series exposure with an active control condition, and more direct contact methods to further test the TTE. Moreover, because outcomes were assessed immediately after exposure, the present studies could not test whether cognitive liberalization effects emerge after consolidation or repetition. Immediate exposure, especially when the interaction is atypical, negative or otherwise cognitively demanding, may temporarily tax cognitive resources; delayed or repeated assessments may therefore provide an even stronger test of whether TTEs unfold over time. Also, we did not measure participants' identification with the protagonist, which may moderate the effectiveness of vicarious contact (Vezzali et al., 2014). Future experimental studies should include such an identification measure. Relatedly, a limitation concerns the use of excerpted scenes from an existing television series, which may provide less narrative continuity than a full‐uninterrupted scene, thereby limiting viewers' immersion or identification with the characters. This may be relevant for interpreting the limited evidence for TTEs, as cognitive liberalization may require more sustained engagement with outgroup perspectives than a brief excerpt‐based exposure.

Second, low reliability in a couple of measures is a critical concern. While this may partly reflect the brevity of the scale, future studies could replicate the design with different and longer scales, and add different types of measures for cognitive processes, such as implicit measures of cognitive flexibility or neurophysiological markers. Third, the study's characterization of high SDO is relative, as even participants classified as high‐SDO scored below the theoretical midpoint of the scale. This suggests a relatively egalitarian sample, which may have attenuated the moderating effects of SDO.

Fourth, manipulation checks suggested that positive interactions with the outgroup were perceived slightly more positively than interactions involving the ingroup, possibly amplifying positivity in the outgroup condition. Future studies should balance portrayals across conditions to isolate intergroup effects. A further limitation is that neither study included a no‐contact baseline condition. Therefore, the present findings cannot determine whether vicarious intergroup contact increases or decreases cognitive liberalization relative to no exposure; rather, they indicate whether cognitive‐liberalization outcomes differed across specific types of vicarious interactions varying in group membership, valence, and prototypicality. Finally, the sample was predominantly composed of young Italian adults, which helped identification with ingroup members in video manipulations, but limited generalizability to broader or more diverse populations. Replicating the study in diverse cultural and demographic contexts may uncover cross‐cultural variations in TTE.

## CONCLUSION

This research underscores the complex, context‐dependent nature of the TTE of intergroup contact. While our results complicate the broader claims of the cognitive liberalization hypothesis, they highlight critical pathways for refining intergroup contact theory and practice. Understanding the roles of prototypicality, valence and individual differences offers a roadmap for interventions that promote creativity, open‐mindedness and cultural deprovincialization. Addressing these nuances can maximize the potential of vicarious contact to foster cognitive and social benefits.

## AUTHOR CONTRIBUTIONS

**Ece Aleyna Demirgünes:** conceptualization, data curation, investigation, methodology, project administration, resources, writing—original draft, writing—review and editing. **Jessica Boin:** conceptualization, data curation, formal analysis, investigation, methodology, software, visualization, writing—original draft; writing—review and editing. **Jasper Van Assche:** writing—original draft, writing—review and editing. **Giulia Fuochi:** conceptualization, data curation, formal analysis, investigation, methodology, project administration, resources, software, supervision, visualization, writing—original draft, writing—review and editing.

## FUNDING INFORMATION

No funding was received.

## CONFLICT OF INTEREST STATEMENT

The authors declared no potential conflicts of interest with respect to the research, authorship and/or publication of this article.

## ETHICS STATEMENT

Both studies were approved by the Ethics Committee for Psychological Research of the University of Padova, protocol numbers #5133 (Study 1) and #5260 (Study 2).

## INFORMED CONSENT

In the data collection of the studies, participants provided their informed consent at the beginning of the online questionnaire.

## Supporting information


**Supplementary Materials**.

## Data Availability

Data, R scripts and preregistrations of these studies are openly available in OSF at https://doi.org/10.17605/OSF.IO/34H2E.

## Footnotes

^1^
We also evaluated – but did not exclude – participants potentially having an immigration background. We asked about their nationality and only 4 participants (1% of 394 participants) in Study 1 and 11 participants (2% of 574 participants) in Study 2 reported having a non‐Italian nationality. We did not exclude them, even though this was an exclusion criterion in the preregistration, because they were a very small percentage of the sample, and because we noticed that the outgroup character of the videos, i.e., Sana, was not presented as having an immigration background, but only as a Muslim girl, thereby justifying the exclusion criterion of Muslim participants. Results do not change if these participants are excluded.

^2^
As robustness checks, and following the preregistration, we repeated the models by controlling for positive and negative direct contact with Muslim people and socio‐demographic variables, i.e., age and gender, and for whether they watched the fourth season of Skam Italia, which is the one having Sana as main character. The inclusion of these controls did not produce any relevant difference in the effects of the manipulations. Moreover, having watched season 4 was almost never associated with the dependent variables, whereas age was occasionally negatively associated with non‐intergroup dependent variables (i.e. RAT and environmental concern in Study 1, MARS in both Study 1 and Study 2), and gender (i.e., being female) was positively associated with the two deprovincialization measures in both studies. Positive and negative direct contact were, respectively, positively and negatively associated with cultural and group deprovincialization (in both studies), while positive contact was positively associated with self‐reported cognitive flexibility (only in Study 1) and with elaboration and originality dimensions of creativity (only in Study 2).
