# Supplementary material for: Experimental tests of the tertiary transfer effect of intergroup contact: Addressing valence, prototypicality and moderators
Source: Br J Soc Psychol. 2026 Jul 8;65(3):e70113. doi: 10.1111/bjso.70113 (PMC13347108; doi:10.1111/bjso.70113)
Supplement: Supplementary file 1 — Supplementary Materials. [file BJSO-65-0-s001.docx]

**Supplementary Materials for the paper “Experimental tests of the tertiary transfer effect of intergroup contact: Addressing valence, prototypicality, and moderators”**

***Summaries of the video manipulations***

In both studies, all videos included a brief contextual introduction to the characters and social setting before the focal interaction segments. Because the clips were taken from the same narrative universe, some characters could appear briefly in introductory or contextual scenes across conditions. However, the focal interaction segments differed according to the intended manipulation: in Study 1, they varied by group membership and valence; in Study 2, they varied by prototypicality and valence.

**Study 1**

***Ingroup Negative Condition***

The video opens with the introduction of key characters: Eva chats and meets with Silvia and Federica. Then, it’s the first time Sana (Muslim – immigrant girl) meets the group, and there’s a sense of unfamiliarity. The girls (Italian girls) seem eager to get to know one another, but this initial gathering sets the stage for an underlying tension. Tensions rise early when Eva argues with another Italian girl, creating an uncomfortable moment. The disagreement is sharp and unresolved, leaving a strained atmosphere between them. This conflict lingers as Eva later meets with the girl group, but the tension remains. Conversations among the group feel uneasy, as the previous argument casts a shadow over their interactions. Later, at a party, the situation escalates further. Eva finds herself in another confrontation with the same Italian girl, and the disagreement spirals out of control. The argument becomes more emotional, with Eva visibly upset and crying, as the tension between them reaches a boiling point. The negative conversation affects the party’s atmosphere, and Eva’s frustration deepens the divide between her and the other girl. The scene closes with the argument unresolved, leaving Eva still grappling with the emotional fallout of her conflict. The tension between her and the Italian girl remains high, with no immediate resolution in sight. The unresolved conflict highlights the strain within the group, affecting their sense of unity.

***Ingroup Positive Condition***

The scene opens with the familiar introduction where Eva meets Sana along with Silvia and Federica. The interaction is friendly and supportive, laying the groundwork for a warm and connected group dynamic. As the story unfolds, Eva is shown in a moment of vulnerability, crying over her boyfriend and feeling desperate. She appears lost, overwhelmed by the situation. In a heartwarming scene, one of her friends comes to comfort her. She listens, reassures her, and offers the support she desperately needs. They hug, and through this comforting moment, Eva begins to feel more at ease, sensing that she is not alone in her struggle. Following this, the girl group gathers around Eva, offering their support. They hug her and provide words of encouragement, reinforcing their friendship and loyalty. It’s a collective show of love and care, giving Eva the emotional backing, she needs. In a more personal moment, Eleonora steps in to cheer Eva up. They’re alone together, and Eleonora lightens the mood by joking, talking, and putting on music. She starts singing along to the music, her playful energy breaking through Eva’s sadness. Slowly but surely, Eva begins to smile, feeling the weight of her emotions lift as Eleonora’s efforts bring her comfort. The scene ends with just the two of them, sharing a moment of connection and friendship, with Eva’s smile signaling a return to a more positive state. Their unity and support for one another are what carry them forward, showing the strength of their friendship.

***Outgroup Negative Condition***

The scene begins with the usual introduction, where Eva introduces Sana to Silvia and Federica. There’s an underlying sense that deeper tensions may surface later on. As the story progresses, Eva and Sana engage in a conversation about relationships between Muslim boys and Italian girls. Eva expresses stereotypical views, making assumptions about cultural differences and implying prejudiced attitudes. In response, Sana counters with frustration: “Because to Muslim boys, Italian girls are basically just seen as easy. This offensive remark adds to the tension, with both Eva`s assumptions and Sana`s reply further straining their relationship and interaction. Later, the girl group, including Sana, is chatting when a more heated conversation arises between Sana and Silvia. The topic of boys and social norms comes up, and Silvia’s views clash with Sana’s perspective as a Muslim. The conversation turns negative, as Sana feels misunderstood and judged for her beliefs, creating a rift between her and Silvia. The tension escalates when Sana learns that the other girl group, along with boys, will be joining them for their upcoming vacation. Sana reacts negatively, feeling blindsided and uncomfortable with the change in plans. This leads to a dispute within the group, with Sana expressing her frustration over the situation, further intensifying the conflict. The disagreement underscores the growing divide between Sana and the group’s views on social dynamics and boundaries. The scene closes on a tense note, with the dispute unresolved and the negative energy between Sana and the rest of the group lingering. The conflict leaves a noticeable strain on their relationships, highlighting the challenges Sana faces in navigating her identity and friendships.

***Outgroup Positive Condition***

The scene opens with the familiar introduction, where Eva introduces Sana to Silvia and Federica. The meeting is warm and friendly, and although the girls come from different backgrounds, the atmosphere is open and inviting, setting the stage for deeper connections. After a lively party, the girls return home to take care of Silvia, who is feeling unwell. They gently help her get into bed, showing a sense of care and friendship. In this intimate moment, Sana unwraps her headscarf for the first time in front of the group, revealing her hair. The girls are quiet but curious, and there’s a peaceful, accepting atmosphere as they see this new side of Sana. The bond between them feels stronger, marked by mutual respect and understanding. They fall asleep together, reflecting a deepening sense of closeness. As Ramadan comes to an end, Sana’s friends visit her house to celebrate Eid. Both the girl group and Sana’s Muslim friends meet and interact, exchanging conversations that reflect a blend of cultures. The environment is warm and inclusive, where the two groups learn from and appreciate one another. Sana, meanwhile, is seen practicing her prayers, peacefully integrating her religious practices into the social gathering. The moment reflects harmony, respect, and understanding between Sana’s faith and her friendships. The final scene shows Sana walking with her girl group in an empowered way. They walk together confidently, unified and supportive of one another. It’s a moment of strength and solidarity, symbolizing how they’ve embraced their differences and grown closer, moving forward as a strong, connected group.

**Study 2**

***Atypical Negative Condition***

The video opens with the introduction of key characters: Eva chats and meetswith Silvia and Federica. Then, it’s the first time Sana (Muslim – immigrant girl) meets the group, and there’s a sense of unfamiliarity. The girls (Italian girls) seem eager to get to know one another, but this initial gathering sets the stage for an underlying tension. As the interactions progress, the tension between Sana and Federica escalates during a private conversation. Federica's remarks, though subtle, strike a nerve, offending Sana. In response, Sana, feeling cornered, retaliates with sharp words. This heated exchange marks the beginning of Sana's growing sense of exclusion within the group. The situation worsens when Sana discovers that the girls have made vacation plans without consulting her, inviting others and making arrangements that clash with her values, particularly when it comes to inviting boys to stay over. This situation highlights her experience of navigating friendships in ways that deviate from her traditional cultural and religious norms and expectations, while still expressing frustration and asserting her identity. This realization deepens the divide, leading to an awkward and negative confrontation between Sana and the rest of the group. The conversation touches on their differing lifestyles and beliefs, with Sana’s identity as a Muslim at the core of the conflict. Feeling isolated and frustrated, Sana records a voice message for the girls. In this deeply emotional message, she expresses her struggles as an immigrant, balancing the difficulty of being accepted as Italian while also confronting the expectations and close-mindedness of her Muslim friends. She confesses her internal conflict never feeling fully "Muslim or Arabic enough" and the constant burden of trying to fit in. Her words are assertive yet heartbroken, revealing her profound emotional pain. She criticizes the community she is part of, but also laments the personal difficulties she faces. It is a powerful and vulnerable moment, where Sana's voice conveys the full weight of her identity struggles and the pain she feels from being caught between two worlds.

***Atypical Positive Condition***

The video begins with Eva introducing Sana to Silvia and Federica. The group is meeting for the first time, and while there’s a sense of curiosity, the interactions feel somewhat tentative. It’s a typical scenario where new acquaintances are being made, and the story moves forward with subtle undercurrents that hint at future developments. After the introduction, Sana is seen watching a basketball game between young men. She observes calmly, keeping her cool throughout the scene. Afterward, she drives a car with confidence, looking relaxed and in control. Later, Sana gets ready for a party. She applies makeup, dances a little in front of the mirror, and carefully puts on her headscarf. This scene feels light and fun, showing her excitement while also embracing her identity but also portrays Sana engaging in behaviors that deviate from traditional expectations for Muslim women, such as dancing while still adhering to her religious identity by wearing her headscarf. At the party, Sana and the girls are at a club, dancing, chatting, and toasting drinks. Sana is enjoying the moment, joining in with the group while maintaining her balance between fun and her own values. The lively energy of the club brings them closer as friends. After the club, the group heads to a sleepover, where they take care of Silvia, who needs some attention after the night out. In this more relaxed setting, Sana removes her headscarf in front of the girls, surprising them. This moment leads to a warm, cozy atmosphere. The girls are accepting and supportive, and the surprise turns into a shared, intimate bond between them. They end up sleeping together in a peaceful, friendly way, solidifying their connection.

***Prototypical Negative Condition***

The scene opens with the familiar introduction of the group. Eva introduces Sana to Silvia and Federica, setting up the initial meeting between the girls. Though they exchange pleasantries, there’s an underlying tension that becomes more evident as the story progresses. As the group settles in, they engage in casual conversation. During this, Silvia begins to share details about a recent sexual encounter, offering explicit descriptions. While the other girls seem unbothered, Sana visibly reacts with discomfort, shifting in her seat and subtly distancing herself. Her unease grows as the conversation continues, creating an air of awkwardness and highlighting a clash between her personal values and the group's openness. Sana`s reactions here reflect her adherence to traditional cultural and religious norms that emphasize modesty and discomfort with explicit discussions. Later, Sana has a conversation with Eva, and the topic shifts to relationships between Muslim boys and Italian girls. Eva expresses stereotypical views, making assumptions about cultural differences and implying prejudiced attitudes. In response, Sana counters with frustration: “Because to Muslim boys, Italian girls are basically just seen as easy. This offensive remark adds to the tension, with both Eva`s assumptions and Sana`s reply further straining their relationship and interaction. The group then gathers at home to discuss their upcoming vacation plans. However, the conversation becomes uncomfortable for Sana as the girls suggest plans that involve inviting boys over and hosting parties, activities that starkly contrast with Sana’s values as a Muslim. The atmosphere turns awkward and negative as Sana struggles to voice her discomfort, feeling increasingly out of place. As the group continues their discussion, Rami, Sana's brother, arrives home unexpectedly. He notices the messy state of the house, with beer bottles scattered around from the girls’ gathering. Rami and Sana exchange a few words about the situation, with Rami giving quick, knowing glances at the girls as they awkwardly try to tidy up the mess. The presence of alcohol and the disarray only further highlights the cultural and personal divide between Sana and her friends.

***Prototypical Positive Condition***

The scene begins with the usual introduction, where Eva introduces Sana to Silvia and Federica. The initial meeting is warm, with curiosity and friendliness in the air. The girls are eager to get to know each other, setting a positive tone for the unfolding events. In a more private moment, Sana explains to Martino (Italian boy) the evolutionary function of homosexuality, offering a thoughtful and insightful perspective on the subject. She also shares an important aspect of her faith, telling Martino that Islam despises insult and offense, emphasizing the values of respect and understanding. Later, the girl group, Sana, and a group of boys gather by the lake to celebrate the end of high school. It’s a lively moment, with the group popping champagne in celebration. While Sana chooses not to drink due to her beliefs, she is fully present and enjoying the company of her friends, showing that she can celebrate alongside them while maintaining her personal values. The scene highlights inclusion and mutual respect, as Sana is part of the joyous moment without feeling excluded. As Ramadan concludes, Sana’s friends come to her house to celebrate Eid with her. In this warm and meaningful gathering, the girl group meets Sana’s Muslim friends. The two groups interact and chat, creating a sense of cultural exchange and mutual appreciation. Sana is also seen practicing her prayers, which exemplifies a behavior traditionally associated with Muslim identity. This portrays the harmonious blending of her faith with her friendships. The final scene features Sana walking alongside her girl group, all walking together in an empowered way. There’s a sense of unity and strength as they move forward together, showcasing the strong bond they’ve built despite their differences. This moment feels uplifting, with each character embracing their individuality while supporting one another.

**Rules for coding AUT categories in the data (with most common examples)**

| ***Verbs/nouns*** | ***Category*** |
| --- | --- |
| Disegnare, colorare, sfumare, annerire, scurire, cerchiare, punto, linea, forme, compasso, dipinto | drawing |
| Scrivere, sottolineare, evidenziare, appuntare, prendere appunti | writing |
| Indicare, comunicare, puntare, esprimere, scegliere, mostrare | pointing/communicating |
| Grattare/si, masticarla, mordicchiarla, scaccolarsi | body entertainment |
| Temperare, appuntire, fare la punta, punta | sharpening |
| Graffiare/si, colpire, uccidere, pungere, punzecchiare, usare come spada | hurting |
| Bucare, incidere, trafiggere, bucare un foglio una gomma, punteruolo | piercing |
| Infilzare, infilare, inficcare, ficcare, pugnalare, | skewering |
| Costruire, creare, inventare, supporto, oggetto (and many more) | crafting |
| Legare i capelli, fermacapelli, fermare i capelli, chignon | hair accessory |
| Giocherellare, giocare, lanciarla, tirarla, roteare, farla cadere, stupire | play |
| Cancellare, gomma, usare la gomma, rimuovere | erasing |
| Suonare, tamburellare, battere, musica, rullare, bacchetta | music |
| Segnalibro, tenere segno nelle pagine | bookmark |
| Misurare, usare come righello, prendere le misure, righello | measuring |
| Rompere(la), spezzar(la), tagliare, bruciarla, rovinare (cose) | breaking |
| Bruciare, appiccare un fuoco, fare un fuoco, fuoco | fire |
| Sporcare, imbrattare, macchiare | dirt |
| Mangiare, prendere il sushi | eating |
| Cucinare, mescolare, usarla come mattarello, stendere la pasta | cooking |
| Truccarsi, matita per occhi, contorno labbra, eyeliner | make-up |
| Spessore, riempire un buco, tappare, chiudere, | filling |
| Infastidire, disturbare, stuzzicare, fare il solletico | bothering |
| Azzerare congegni elettronici | electronics |
| Spostare, fare leva, sollevare, spingere | moving |
| Guardarla, curarla, studiarla, dormirci insieme | care |
| Spingere, premere, aprire una confezione/ una lattina, stappare | pushing |
| Scavare, piantare, piante | digging |
| Arrotolare cassetta, aggiustare, pulire | fixing |
| Regalare, prestare, passare a qualcuno | gift/lending |
| Riporla, metterla in un astuccio, metterla via, buttare, nascondere/la | put away |
| Acquistare, comprare | buying |
| Vendere | selling |
| Perderla | losing |
| Arrotolare/arricciare i capelli | hairdo |
| Toccare, tastare, sfiorare, accarezzare | touching |
| Prendere, raccogliere, rubare | taking |
| Fermare oggetti, fermaporta, bloccare, sostenere, fermacarte | stopping |
| Lisciare, appianare, affilare, piegare | smoothing |
| Farla cadere per perdere tempo, passare il tempo, rotolare, passatempo | distraction |
| Collezionare, fare una collezione | collect |

**Functions’ originality is evaluated according to their rareness, i.e., they are original if:**

1) They appear in a limited amount of cases (< 10%)

**AND**

2) They are different from other functions (e.g., punteggiare may appear rarely, but it is similar to other writing functions such as tratteggiare), even though they belong to common categories

e.g.: dardo, freccette: category play, but very rare functions.

**Attention checks and exclusion criterion**

Attention checks consisted of four true-or-false questions asking participants whether or not they saw a situation in the video (e.g., girls going out to a party, the name of one of the characters), and they varied depending on the experimental condition. Each correct answer was scored as 1 and each error (i.e., incorrectly endorsing a false statement or failing to recognize a true statement) was scored as 0, resulting in a maximum possible score of 4. Participants who scored less than 3 (i.e., made more than one error) were excluded from the analysis, as this was considered evidence of insufficient attention to the experimental materials.

***Manipulation checks results in Study 1***

We conducted mean comparisons to analyze differences among conditions in the manipulation check questions. As for emotions and perceived valence of the video manipulations, two-way ANOVAs revealed that the videos showing positive contact were rated as more positive (*F*(1, 390) = 402.51, *p* < .001, *η_p_*² = .50), and less negative (*F*(1, 570) = 389.12, *p* < .001, *η_p_*² = .49) than those showing negative contact. These videos also elicited significantly more positive emotions (*F*(1, 389) = 242.36, *p* < .001, *η_p_*² = .38) and less negative emotions (*F*(1, 390) = 236.68, *p* < .001, *η_p_*² = .37) compared to the videos showing negative contact, confirming the effectiveness of the valence manipulation. Additionally, a main effect of group membership also emerged: videos portraying contact with the outgroup were rated as more positive (*F*(1, 390) = 23.69, *p* < .001, *η_p_*² = .06) and less negative (*F*(1, 389) = 33.09, *p* < .001, *η_p_*² = .08), and they evoked more positive (*F*(1, 389) = 15.10, *p* < .001, *η_p_*² = .04) and fewer negative emotions (*F*(1, 390) = 16.97, *p* < .001, *η_p_*² = .04) compared to those showing contact with the ingroup. As for the perceived valence of the situation, a statistically significant interaction between valence and group membership emerged for both positive (*F*(1, 390) = 5.95, *p* = .015, *η_p_*² = .02) and negative valence (*F*(1, 389) = 11.03, *p* < .001, *η_p_*² = .03). Results of Tukey-adjusted pairwise comparisons of estimated marginal means showed that for the positive valence, the *ingroup negative* (*M* = 2.36, *SD* = 1.27) and *outgroup negative* (*M* = 2.68, *SD* = 1.06) videos were not different from one another (*t* (390) = -1.784, *p* = .075, *d* = 0.27). However, the *ingroup positive* video (*M* = 4.58, *SD* = 1.40) was rated more positively (*t* (390) = -12.23, *p* < .001, *d* = 1.66) than *ingroup negative* (*M* = 2.36, *SD* = 1.27). Similarly, the *outgroup positive* (*M* = 5.52, *SD* = 1.06) video was perceived as more positive (*t* (390) = -15.78, *p* < .001, *d* = 2.36) than the *outgroup negative* video (*M* = 2.68, *SD* = 1.33), but also more positive (*t* (390) = -5.14, *p* < .001, *d* = 0.69) than the *ingroup positive* video (*M* = 4.58, *SD* = 1.40). As regards to negative valence, no difference was found between the *ingroup negative* (*M* = 5.56, *SD* = 1.15) and *outgroup negative* (*M* = 5.25, *SD* = 1.25) videos (*t* (389) = 1.17, *p* = .084, *d* = 0.26). The *ingroup positive* (*M* = 3.47, *SD* = 1.57) video was perceived as less negative (*t* (389) = -11.29, *p* <.001. *d* = 1.52) than the *ingroup negative* (*M* = 5.56, *SD* = 1.15) video, and more negatively (*t* (389) = 6.48, *p* <.001, *d* = 0.87) than the *outgroup positive* video (*M* = 2.26, *SD* = 1.19). The *outgroup positive* (*M* = 2.26, *SD* = 1.19) video was perceived as less negative (*t* (389) =16.22, *p* < .001, *d* = 0.45) than the *outgroup negative* (*M* = 5.25, *SD* = 1.25) videos.

A statistically significant valence x group membership interaction also emerged for both positive (*F*(1, 390) = 7.44, *p* = .007, *η_p_*² = .02) and negative emotions (*F*(1, 389) = 6.50, *p* = .011, *η_p_*² = .02), with Tukey-adjusted pairwise comparisons of estimated marginal means suggesting that the *ingroup negative* (positive emotions: *M* = 2.62, *SD* = 1.30; negative emotions: *M* = 5.17, *SD* = 1.45) and *outgroup negative* (positive emotions: *M* = 2.81, *SD* =1.46; negative emotions: *M* = 4.96, *SD* = 1.52) conditions were not different from one another (*t* (389) = -0.997, *p* = .319, *d* = 0.14 for positive emotions; *t* (390) = 1.047, *p* = .296, *d* = 0.53 for negative emotions). However, the *ingroup positive* video (positive emotions: *M* = 4.39, *SD* = 1.33; negative emotions: *M* = 3.36, *SD* = 1.50) elicited more positive (*t* (389) = -9.03, *p* <.001, *d* = 1.35) and less negative (*t* (390) = 8.76, *p* <.001, *d* = 1.23) emotions than the *ingroup negative* video (positive emotions: *M* = 2.62, *SD* = 1.30; negative emotions: *M* = 5.17, *SD* = 1.45), but less positive (*t* (389) = -4.54, *p* <.001, *d* = 0.72) and more negative emotions (*t* (390) = 4.83, *p* <.001, *d* = 0.71) than the *outgroup positive* video (positive emotions: *M* = 5.30, *SD* = 1.20; negative emotions: *M* = 2.36, *SD* = 1.29). The *outgroup positive* video evoked more positive (*t* (389) = -12.69, *p* <.001, *d* = 1.86) and less negative (*t* (390) = 12.69, *p* <.001, *d* = 1.84) emotions than the *outgroup negative* (positive emotions: *M* = 2.81, *SD* =1.46; negative emotions: *M* = 4.96, *SD* = 1.52).

Finally, as regard to the plausibility and the difficulty in understanding the videos, the two-way ANOVAs revealed a main effect of the group membership (plausibility: *F*(1, 390) = 11.55, *p* = .013, *η_p_*² = .02; difficulty: *F*(1, 390) = 10.77, *p* = .048, *η_p_*² = .01). Participants thought that the situations represented in the videos were slightly more plausible in the *outgroup* conditions (*M* = 5.69, *SD* = 1.27) than in the *ingroup* conditions (*M* = 5.36, *SD* = 1.45), and perceived the interactions with the *ingroup* (*M* = 2.84, *SD* = 1.72) as more difficult to understand than the interactions with the *outgroup* (*M* = 2.49, *SD* = 1.58).

***Manipulation checks results in Study 2***

We analyzed differences among conditions in the manipulation check questions through a series of mean comparisons. First, we verified through a one-way ANOVA that participants were aware of being in a different group than Sana’s in the same way across all the conditions, and we found so, *F*(3, 569) = 1.87, *p* = .314, *η_p_*² = .01 (mean values between 5.06 for *atypical positive* and 5.41 for *prototypical negative*). Second, through a two-way ANOVA, we confirmed that Sana was perceived as more typical of her group in the conditions with low semantic distance, i.e., higher prototypicality, than with higher semantic distance, *F*(1, 570) = 22.35, *p* < .001, *η_p_*² = .04 (*prototypical negative*: *M* = 4.65, *SD* = 1.46; *prototypical positive*: *M* = 5.01, *SD* = 1.38; *atypical negative*: *M* = 4.18, *SD* = 1.43; *atypical positive*: *M* = 4.34, *SD* = 1.61), thereby providing support of the effectiveness of the semantic distance manipulation. Sana was also perceived slightly more prototypical when contact valence was negative compared to positive, *F*(1, 570) = 4.52, *p* = .034, *η_p_*² = .01, consistent with the valence-salience effect (Paolini et al., 2010).

As for emotions and perceived valence of the video manipulations, through two-way ANOVAs, we verified that the videos portraying positive contact were perceived as more positive (*F*(1, 570) = 612.05, *p* < .001, *η_p_*² = .52), and less negative (*F*(1, 570) = 473.51, *p* < .001, *η_p_*² = .45), and that they elicited more positive emotions (*F*(1, 570) = 347.43, *p* < .001, *η_p_*² = .38) and less negative emotions (*F*(1, 570) = 396.13, *p* < .001, *η_p_*² = .41) compared to the videos portraying negative contact, thereby proving the effectiveness of the valence manipulation. However, some effects of semantic distance also emerged: regarding the perceived positive valence of the videos, the two manipulations had a statistically significant interaction (*F*(1, 570) = 9.63, *p* = .002, *η_p_*² = .02), with Tukey-adjusted pairwise comparisons of estimated marginal means suggesting that the *prototypical positive* (*M* = 4.77, *SD* = 1.22) and *atypical positive* (*M* = 4.64, *SD* = 1.27) videos did not yield any difference (*t* (570) = 0.128, *p* = .353, *d* = 0.10), whereas the *atypical negative* video (*M* = 2.50, *SD* = 1.28) was perceived more positively (*t* (570) = -0.481, *p* = .001, *d* = 0.44) than the *prototypical negative* video (*M* = 2.02, *SD* = 0.84). Regarding positive emotions, the two manipulations also had a statistically significant interaction (*F*(1, 570) = 9.85, *p* = .002, *η_p_*² = .02), with Tukey-adjusted pairwise comparisons of estimated marginal means showing that the *prototypical positive* (*M* = 4.77, *SD* = 1.32) and *atypical positive* (*M* = 4.58, *SD* = 1.56) videos did not yield any difference (*t* (570) = 0.184, *p* = .243, *d* = 0.13), whereas the *atypical negative* video (*M* = 2.83, *SD* = 1.35) elicited more positive emotions (*t* (570) = -0.519, *p* = .001, *d* = 0.13) than the *prototypical negative* video (*M* = 2.32, *SD* = 1.06), consistent with what we found for the perceived positive valence of the videos. Regarding negative emotions, a main effect of semantic distance emerged (*F*(1, 570) = 14.96, *p* < .001, *η_p_*² = .03), with the *prototypical* conditions (*negative*: *M* = 5.62, *SD* = 0.88; *positive*: *M* = 3.36, *SD* = 1.53) eliciting more negative emotions than the respective *atypical* conditions (*negative*: *M* = 5.17, *SD* = 1.35; *positive*: *M* = 2.93, *SD* = 1.49).

Lastly, we found that participants thought that the situations represented in the videos were equally plausible across conditions (one-way ANOVA: *F*(3, 570) = 0.911, *p* = .435, *η_p_*² = .00, mean values between 5.62 and 5.84), but that both the *atypical* videos were more difficult to understand than the *prototypical* videos (two-way ANOVA, effect of semantic distance: *F*(1, 570) = 26.66, *p* < .001, *η_p_*² = .04), even though difficulty in understanding the videos was overall very low, with mean values between 1.82 (*prototypical negative*) and 2.47 (*atypical negative*).

**Table S1. Means, standard deviations, and intercorrelations among variable in Study 1**

|  | *M* | *SD* | *1.* | *2.* | *3.* | *4.* | *5.* | *6.* | *7.* | *8.* | *9.* | *10.* | *11.* | *12.* | *13.* | *14.* |
| --- | --- | --- | --- | --- | --- | --- | --- | --- | --- | --- | --- | --- | --- | --- | --- | --- |
| 1.Cognitive Flexibility | 4.96 | 0.79 |  |  |  |  |  |  |  |  |  |  |  |  |  |  |
| 2.Fluency– AUT | 7.77 | 4.06 | .07 |  |  |  |  |  |  |  |  |  |  |  |  |  |
| 3.Elaboration– AUT | 9.19 | 5.21 | .09 | .90^***^ |  |  |  |  |  |  |  |  |  |  |  |  |
| 4.Flexibility– AUT | 4.85 | 2.57 | .01 | .83^***^ | .78^***^ |  |  |  |  |  |  |  |  |  |  |  |
| 5.Originality– AUT | 0.35 | 0.65 | .04 | .48^***^ | .59^***^ | .52^***^ |  |  |  |  |  |  |  |  |  |  |
| 6.MARS | 3.30 | 1.23 | -.00 | .18^***^ | .17^**^ | .16^**^ | .07 |  |  |  |  |  |  |  |  |  |
| 7.RAT | 1.77 | 1.38 | .04 | .18^***^ | .20^***^ | .21^***^ | .16^**^ | .16^**^ |  |  |  |  |  |  |  |  |
| 8.Cultural Deprovincialization | 6.03 | 0.95 | .21^***^ | .08 | .08 | .05 | .07 | .02 | .00 |  |  |  |  |  |  |  |
| 9.Group Deprovincialization | 6.35 | 0.81 | .15^**^ | .20^***^ | .18^***^ | .17^***^ | .13^**^ | .03 | .05 | .47^***^ |  |  |  |  |  |  |
| 10.Environmental Concern | 6.19 | 0.93 | .03 | .12^*^ | .09 | .11^*^ | .02 | .01 | .07 | .24^***^ | .22^***^ |  |  |  |  |  |
| 11.SDO | 1.95 | 0.93 | -.07 | -.05 | -.09 | -.04 | -.04 | -.05 | -.13^**^ | -.40^***^ | -.33^***^ | -.24^***^ |  |  |  |  |
| 12.NFC | 4.22 | 0.86 | -.15^**^ | -.12^*^ | -.11^*^ | -.14^**^ | -.05 | -.16^**^ | -.07 | -.15^**^ | -.07 | -.03 | .13^*^ |  |  |  |
| 13.Curiosity | 5.29 | 0.93 | .41^***^ | .13^**^ | .15^**^ | .08 | .08 | .08 | .11^*^ | .39^***^ | .25^***^ | .11^*^ | -.15^**^ | -.23^***^ |  |  |
| 14.Positive direct contact | 2.69 | 1.13 | .10^*^ | .08 | .07 | .08 | .04 | -.01 | .04 | .24^***^ | .22^***^ | -.05 | -.07 | -.09 | .20^***^ |  |
| 15.Negative direct contact | 1.42 | 0.66 | -.03 | .06 | .02 | .04 | .03 | .01 | -.05 | -.07 | -.06 | -.00 | .19^***^ | -.05 | .05 | .26^***^ |

*Note.* ^*^ *p <* .05, ^**^ *p <* .01, ^***^ *p <* .001.

**Table S2**. **Manipulation Check Results of Study 1**

| **Manipulation Check** | ***F*** | ***df*** | ***p*** | ***ηp²*** |
| --- | --- | --- | --- | --- |
|  |  |  |  |  |
| Perceived positive valence of video — Valence main effect | 402.51 | 1, 390 | < .001 | .50 |
| Perceived positive valence of video — Group membership main effect | 23.69 | 1, 390 | < .001 | .06 |
| Perceived positive valence of video — Valence × Group interaction | 5.95 | 1, 390 | .015 | .02 |
| Perceived negative valence of video — Valence main effect | 389.12 | 1, 570 | < .001 | .49 |
| Perceived negative valence of video — Group membership main effect | 33.09 | 1, 389 | < .001 | .08 |
| Perceived negative valence of video — Valence × Group interaction | 11.03 | 1, 389 | < .001 | .03 |
| Positive emotions experienced — Valence main effect | 242.36 | 1, 389 | < .001 | .38 |
| Positive emotions — Group membership main effect | 15.10 | 1, 389 | < .001 | .04 |
| Positive emotions — Valence × Group interaction | 7.44 | 1, 390 | .007 | .02 |
| Negative emotions experienced — Valence main effect | 236.68 | 1, 390 | < .001 | .37 |
| Negative emotions — Group membership main effect | 16.97 | 1, 390 | < .001 | .04 |
| Negative emotions — Valence × Group interaction | 6.50 | 1, 389 | .011 | .02 |
| Plausibility of video — Group membership main effect | 11.55 | 1, 390 | .013 | .02 |
| Difficulty understanding video — Group membership main effect | 10.77 | 1, 390 | .048 | .01 |

Note. Valence = positive vs. negative intergroup contact video; Group membership = ingroup vs. outgroup target. **Unless otherwise indicated, tests are two-way ANOVAs with factors Valence and Group membership.**

**Table S3. The effects of the manipulations (Group Membership and Valence) on Cultural and Group Deprovincialization, and Environmental Concern, Study 1**

|  | ***Cultural Deprovincialization*** | | | | | ***Group Deprovincialization*** | | | | | ***Environmental concern*** | | | | |
| --- | --- | --- | --- | --- | --- | --- | --- | --- | --- | --- | --- | --- | --- | --- | --- |
|  | *b* | *SE* | β | *95% CI* | *p* | *b* | *SE* | β | *95% CI* | *p* | *b* | *SE* | β | *95% CI* | *p* |
| Intercept | 6.05 | 0.09 | -.00 | 5.86 – 6.23 | <.001 | 6.34 | 0.08 | -.00 | 6.18 – 6.50 | <.001 | 6.09 | 0.09 | .00 | 5.91 – 6.27 | <.001 |
| Outgroup | -0.11 | 0.13 | -.02 | -0.38 – 0.15 | .394 | -0.15 | 0.11 | -.06 | -0.38 – 0.07 | .176 | 0.21 | 0.13 | .04 | -0.05 – 0.46 | .117 |
| Positive | -0.00 | 0.14 | .04 | -0.27 – 0.26 | .976 | 0.11 | 0.12 | .10 | -0.12 – 0.34 | .332 | 0.13 | 0.13 | -.00 | -0.13 – 0.40 | .313 |
| Outgroup × Positive | 0.17 | 0.19 | .04 | -0.21 – 0.54 | .389 | 0.11 | 0.16 | .03 | -0.22 – 0.43 | .518 | -0.28 | 0.19 | -.07 | -0.64 – 0.09 | .143 |
| R² | 0.004 | | | | | 0.015 | | | | | 0.007 | | | | |

Note. Outgroup (0 = ingroup interaction, 1 = outgroup interaction) = effect of watching the video including an interaction with the outgroup compared to an interaction with the ingroup (Group Membership factor); Positive (0 = negative interaction, 1 = positive interaction) = effect of watching the video including positive contact compared to negative (Valence factor).

**Table S4. The effects of the manipulations (Group Membership and Valence) on MARS, and RAT, Study 1**

|  | ***MARS*** | | | | | ***RAT*** | | | | |
| --- | --- | --- | --- | --- | --- | --- | --- | --- | --- | --- |
|  | *b* | *SE* | β | *95% CI* | *p* | *b* | *SE* | β | *95% CI* | *p* |
| Intercept | 3.31 | 0.12 | .00 | 3.08 – 3.55 | <.001 | 1.75 | 0.13 | -.00 | 1.49 – 2.02 | <.001 |
| Outgroup | 0.01 | 0.17 | -.06 | -0.33 – 0.35 | .944 | 0.09 | 0.19 | .09 | -0.29 – 0.48 | .626 |
| Positive | 0.14 | 0.18 | -.02 | -0.21 – 0.48 | .440 | -0.20 | 0.20 | -.02 | -0.59 – 0.19 | .305 |
| Outgroup × Positive | -0.34 | 0.25 | -.07 | -0.83 – 0.14 | .166 | 0.30 | 0.28 | .05 | -0.25 – 0.84 | .287 |
| R² | 0.009 | | | | | 0.011 | | | | |

Note. Outgroup (0 = ingroup interaction, 1 = outgroup interaction) = effect of watching the video including an interaction with the outgroup compared to an interaction with the ingroup (Group Membership factor); Positive (0 = negative interaction, 1 = positive interaction) = effect of watching the video including positive contact compared to negative (Valence factor).

**Table S5. The effects of the manipulations (Group Membership and Valence) on Cognitive Flexibility (CF) items, Study 1**

|  | ***CF item 1*** | | | | | ***CF item 2*** | | | | |
| --- | --- | --- | --- | --- | --- | --- | --- | --- | --- | --- |
|  | *b* | *SE* | β | *95% CI* | *p* | *b* | *SE* | β | *95% CI* | *p* |
| Intercept | 4.71 | 0.12 | -.00 | 4.47 – 4.95 | <.001 | 5.62 | 0.17 | -.00 | 5.28 – 5.95 | <.001 |
| Outgroup | 0.13 | 0.17 | .10 | -0.21 – 0.47 | .461 | -0.03 | 0.24 | .03 | -0.51 – 0.45 | .890 |
| Positive | -0.11 | 0.18 | .00 | -0.46 – 0.24 | .540 | 0.02 | 0.25 | .05 | -0.47 – 0.51 | .930 |
| Outgroup × Positive | 0.23 | 0.25 | .05 | -0.26 – 0.72 | .352 | 0.32 | 0.35 | .05 | -0.38 – 1.01 | .370 |
| R² | 0.012 | | | | | 0.006 | | | | |

Note. Outgroup (0 = ingroup interaction, 1 = outgroup interaction) = effect of watching the video including an interaction with the outgroup compared to an interaction with the ingroup (Group Membership factor); Positive (0 = negative interaction, 1 = positive interaction) = effect of watching the video including positive contact compared to negative (Valence factor).

**Table S5 (continued). The effects of the manipulations (Group Membership and Valence) on Cognitive Flexibility (CF) items, Study 1**

|  | ***CF item 3*** | | | | | ***CF item 4*** | | | | |
| --- | --- | --- | --- | --- | --- | --- | --- | --- | --- | --- |
|  | *b* | *SE* | β | *95% CI* | *p* | *b* | *SE* | β | *95% CI* | *p* |
| Intercept | 4.31 | 0.16 | -.00 | 4.00 – 4.61 | <.001 | 4.76 | 0.11 | -.00 | 4.53 – 4.99 | <.001 |
| Outgroup | -0.19 | 0.22 | -.04 | -0.62 – 0.25 | .406 | 0.18 | 0.17 | .10 | -0.15 – 0.50 | .281 |
| Positive | 0.31 | 0.23 | .12 | -0.14 – 0.75 | .177 | 0.20 | 0.17 | .10 | -0.13 – 0.53 | .244 |
| Outgroup × Positive | 0.13 | 0.32 | .02 | -0.50 – 0.76 | .685 | 0.10 | 0.24 | .02 | -0.37 – 0.56 | .681 |
| R² | 0.015 | | | | | 0.021 | | | | |

Note. Outgroup (0 = ingroup interaction, 1 = outgroup interaction) = effect of watching the video including an interaction with the outgroup compared to an interaction with the ingroup (Group Membership factor); Positive (0 = negative interaction, 1 = positive interaction) = effect of watching the video including positive contact compared to negative (Valence factor).

**Table S6. The effects of the manipulations (Group Membership and Valence) on AUT dimensions, Study 1**

|  | ***Fluency – AUT*** | | | | | ***Elaboration – AUT*** | | | | | ***Originality – AUT*** | | | | |
| --- | --- | --- | --- | --- | --- | --- | --- | --- | --- | --- | --- | --- | --- | --- | --- |
|  | *b* | *SE* | β | *95% CI* | *p* | *b* | *SE* | β | *95% CI* | *p* | *b* | *SE* | β | *95% CI* | *p* |
| Intercept | 7.59 | 0.40 | .00 | 6.81 – 8.37 | <.001 | 9.01 | 0.51 | .00 | 8.01 – 10.01 | <.001 | 0.32 | 0.06 | .00 | 0.20 – 0.45 | <.001 |
| Outgroup | 0.13 | 0.57 | -.06 | -0.99 – 1.26 | .815 | -0.05 | 0.73 | -.05 | -1.49 – 1.39 | .945 | 0.04 | 0.09 | -.03 | -0.14 – 0.22 | .633 |
| Positive | 0.99 | 0.59 | .04 | -0.16 – 2.14 | .091 | 1.01 | 0.75 | .05 | -0.46 – 2.48 | .177 | 0.08 | 0.09 | .00 | -0.10 – 0.27 | .373 |
| Outgroup × Positive | -1.32 | 0.82 | -.08 | -2.94 – 0.30 | .110 | -1.02 | 1.05 | -.05 | -3.09 – 1.05 | .333 | -0.16 | 0.13 | -.06 | -0.42 – 0.09 | .213 |
| R² | 0.012 | | | | | 0.007 | | | | | 0.005 | | | | |

Note. Outgroup (0 = ingroup interaction, 1 = outgroup interaction) = effect of watching the video including an interaction with the outgroup compared to an interaction with the ingroup (Group Membership factor); Positive (0 = negative interaction, 1 = positive interaction) = effect of watching the video including positive contact compared to negative (Valence factor).

**Table S7. The moderation of SDO in the models for RAT and Cultural Deprovincialization, Study 1**

|  | ***RAT*** | | | | | ***Cultural Deprovincialization*** | | | | |
| --- | --- | --- | --- | --- | --- | --- | --- | --- | --- | --- |
|  | *b* | *SE* | β | *95% CI* | *p* | *b* | *SE* | β | *95% CI* | *p* |
| Intercept | 1.96 | 0.30 | -.00 | 1.37 – 2.54 | <.001 | 6.78 | 0.19 | -.00 | 6.40 – 7.16 | <.001 |
| Outgroup | 0.68 | 0.44 | .08 | -0.18 – 1.54 | .122 | 0.05 | 0.28 | -.03 | -0.51 – 0.60 | .870 |
| Positive | -0.58 | 0.48 | -.04 | -1.52 – 0.35 | .222 | -0.07 | 0.31 | -.01 | -0.67 – 0.54 | .831 |
| SDO | -0.10 | 0.13 | -.12 | -0.35 – 0.16 | .449 | -0.35 | 0.08 | -.40 | -0.52 – -0.19 | <.001 |
| Outgroup × Positive | 0.44 | 0.66 | .04 | -0.86 – 1.74 | .505 | 0.18 | 0.43 | .03 | -0.65 – 1.02 | .669 |
| Outgroup × SDO | -0.28 | 0.19 | -.12 | -0.65 – 0.09 | .141 | -0.08 | 0.12 | -.05 | -0.32 – 0.16 | .538 |
| Positive × SDO | 0.19 | 0.22 | .05 | -0.25 – 0.63 | .396 | -0.00 | 0.14 | -.01 | -0.29 – 0.28 | .972 |
| Outgroup × Positive × SDO | -0.12 | 0.31 | -.01 | -0.73 – 0.49 | .697 | -0.04 | 0.20 | -.01 | -0.44 – 0.35 | .834 |
| R² | 0.040 | | | | | 0.158 | | | | |

Note. Outgroup (0 = ingroup interaction, 1 = outgroup interaction) = effect of watching the video including an interaction with the outgroup compared to an interaction with the ingroup (Group Membership factor); Positive (0 = negative interaction, 1 = positive interaction) = effect of watching the video including positive contact compared to negative (Valence factor). SDO = Social Dominance Orientation, a measure of preference for hierarchical versus egalitarian social relations.

**Table S8. The moderation of SDO in the models for Group Deprovincialization, and Environmental Concern dimensions, Study 1**

|  | ***Group Deprovincialization*** | | | | | ***Environmental concern*** | | | | |
| --- | --- | --- | --- | --- | --- | --- | --- | --- | --- | --- |
|  | *b* | *SE* | β | *95% CI* | *p* | *b* | *SE* | β | *95% CI* | *p* |
| Intercept | 6.83 | 0.17 | -.00 | 6.50 – 7.16 | <.001 | 6.71 | 0.20 | -.00 | 6.32 – 7.09 | <.001 |
| Outgroup | 0.07 | 0.25 | -.07 | -0.41 – 0.55 | .775 | -0.17 | 0.29 | .02 | -0.74 – 0.40 | .563 |
| Positive | 0.10 | 0.27 | .06 | -0.42 – 0.63 | .697 | -0.03 | 0.31 | -.04 | -0.64 – 0.59 | .928 |
| SDO | -0.23 | 0.07 | -.33 | -0.38 – -0.09 | .001 | -0.30 | 0.08 | -.26 | -0.46 – -0.13 | .001 |
| Outgroup × Positive | 0.02 | 0.37 | .01 | -0.70 – 0.75 | .947 | 0.32 | 0.44 | -.08 | -0.54 – 1.17 | .468 |
| Outgroup × SDO | -0.11 | 0.11 | -.05 | -0.32 – 0.10 | .317 | 0.18 | 0.13 | .01 | -0.07 – 0.43 | .150 |
| Positive × SDO | -0.01 | 0.12 | -.01 | -0.26 – 0.23 | .914 | 0.05 | 0.15 | -.05 | -0.23 – 0.34 | .711 |
| Outgroup × Positive × SDO | 0.01 | 0.17 | -.00 | -0.34 – 0.35 | .967 | -0.31 | 0.20 | -.08 | -0.72 – 0.09 | .126 |
| R² | 0.128 | | | | | 0.078 | | | | |

Note. Outgroup (0 = ingroup interaction, 1 = outgroup interaction) = effect of watching the video including an interaction with the outgroup compared to an interaction with the ingroup (Group Membership factor); Positive (0 = negative interaction, 1 = positive interaction) = effect of watching the video including positive contact compared to negative (Valence factor). SDO = Social Dominance Orientation, a measure of preference for hierarchical versus egalitarian social relations.

**Table S9. The moderation of SDO in the models for AUT dimensions, Study 1**

|  | ***Fluency – AUT*** | | | | | ***Flexibility – AUT*** | | | | |
| --- | --- | --- | --- | --- | --- | --- | --- | --- | --- | --- |
|  | *b* | *SE* | β | *95% CI* | *p* | *b* | *SE* | β | *95% CI* | *p* |
| Intercept | 6.98 | 0.88 | .00 | 5.25 – 8.71 | <.001 | 4.22 | 0.55 | .01 | 3.13 – 5.31 | <.001 |
| Outgroup | 2.03 | 1.29 | -.06 | -0.51 – 4.57 | .118 | 1.50 | 0.82 | -.07 | -0.10 – 3.11 | .066 |
| Positive | 1.51 | 1.40 | .03 | -1.25 – 4.26 | .283 | 0.69 | 0.88 | .07 | -1.05 – 2.43 | .436 |
| SDO | 0.29 | 0.38 | -.06 | -0.45 – 1.04 | .438 | 0.18 | 0.24 | -.03 | -0.29 – 0.65 | .449 |
| Outgroup × Positive | -2.40 | 1.95 | -.08 | -6.23 – 1.42 | .217 | -1.27 | 1.23 | -.11 | -3.68 – 1.15 | .302 |
| Outgroup × SDO | -0.91 | 0.56 | -.09 | -2.01 – 0.19 | .104 | -0.67 | 0.35 | -.12 | -1.36 – 0.03 | .059 |
| Positive × SDO | -0.33 | 0.65 | .01 | -1.61 – 0.96 | .617 | 0.12 | 0.41 | .03 | -0.69 – 0.94 | .762 |
| Outgroup × Positive × SDO | 0.54 | 0.92 | .05 | -1.26 – 2.34 | .553 | 0.07 | 0.58 | .01 | -1.07 – 1.20 | .908 |
| R² | 0.018 | | | | | 0.034 | | | | |

Note. Outgroup (0 = ingroup interaction, 1 = outgroup interaction) = effect of watching the video including an interaction with the outgroup compared to an interaction with the ingroup (Group Membership factor); Positive (0 = negative interaction, 1 = positive interaction) = effect of watching the video including positive contact compared to negative (Valence factor). SDO = Social Dominance Orientation, a measure of preference for hierarchical versus egalitarian social relations.

**Table S9 (continued). The moderation of SDO in the models for AUT dimensions, Study 1**

|  | ***Elaboration – AUT*** | | | | | ***Originality – AUT*** | | | | |
| --- | --- | --- | --- | --- | --- | --- | --- | --- | --- | --- |
|  | *b* | *SE* | β | *95% CI* | *p* | *b* | *SE* | β | *95% CI* | *p* |
| Intercept | 8.76 | 1.13 | .00 | 6.53 – 10.99 | <.001 | 0.24 | 0.14 | .00 | -0.04 – 0.52 | .090 |
| Outgroup | 2.14 | 1.67 | -.05 | -1.13 – 5.42 | .199 | 0.32 | 0.21 | -.02 | -0.09 – 0.73 | .128 |
| Positive | 2.02 | 1.81 | .04 | -1.53 – 5.57 | .264 | 0.29 | 0.23 | -.01 | -0.16 – 0.73 | .204 |
| SDO | 0.12 | 0.49 | -.09 | -0.84 – 1.08 | .807 | 0.04 | 0.06 | -.05 | -0.08 – 0.16 | .514 |
| Outgroup × Positive | -3.17 | 2.51 | -.05 | -8.10 – 1.77 | .208 | -0.49 | 0.31 | -.07 | -1.10 – 0.13 | .121 |
| Outgroup × SDO | -1.05 | 0.72 | -.06 | -2.47 – 0.36 | .145 | -0.13 | 0.09 | -.04 | -0.31 – 0.05 | .144 |
| Positive × SDO | -0.60 | 0.84 | .01 | -2.26 – 1.06 | .477 | -0.10 | 0.11 | -.02 | -0.31 – 0.11 | .334 |
| Outgroup × Positive × SDO | 1.08 | 1.18 | .06 | -1.24 – 3.40 | .361 | 0.15 | 0.15 | .05 | -0.14 – 0.44 | .298 |
| R² | 0.016 | | | | | 0.013 | | | | |

Note. Outgroup (0 = ingroup interaction, 1 = outgroup interaction) = effect of watching the video including an interaction with the outgroup compared to an interaction with the ingroup (Group Membership factor); Positive (0 = negative interaction, 1 = positive interaction) = effect of watching the video including positive contact compared to negative (Valence factor). SDO = Social Dominance Orientation, a measure of preference for hierarchical versus egalitarian social relations.

**Table S10. The moderation of NFC in the models for MARS, and RAT, Study 1**

|  | ***MARS*** | | | | | ***RAT*** | | | | |
| --- | --- | --- | --- | --- | --- | --- | --- | --- | --- | --- |
|  | *b* | *SE* | β | *95% CI* | *p* | *b* | *SE* | β | *95% CI* | *p* |
| Intercept | 3.30 | 0.59 | -.01 | 2.14 – 4.47 | <.001 | 1.83 | 0.68 | -.00 | 0.50 – 3.16 | .007 |
| Outgroup | 0.69 | 0.86 | -.05 | -0.99 – 2.37 | .420 | 0.56 | 0.98 | .09 | -1.37 – 2.48 | .570 |
| Positive | 2.35 | 0.84 | -.03 | 0.70 – 4.00 | .005 | 0.44 | 0.96 | -.02 | -1.44 – 2.33 | .645 |
| NFC | 0.00 | 0.14 | -.15 | -0.27 – 0.27 | .986 | -0.02 | 0.16 | -.08 | -0.32 – 0.29 | .906 |
| Outgroup × Positive | -2.33 | 1.22 | -.06 | -4.74 – 0.08 | .058 | -0.01 | 1.40 | .06 | -2.77 – 2.74 | .992 |
| Outgroup × NFC | -0.16 | 0.20 | .03 | -0.55 – 0.23 | .418 | -0.11 | 0.22 | -.02 | -0.55 – 0.33 | .630 |
| Positive × NFC | -0.54 | 0.20 | -.10 | -0.92 – -0.15 | .006 | -0.16 | 0.22 | -.04 | -0.60 – 0.28 | .479 |
| Outgroup × Positive × NFC | 0.48 | 0.28 | .08 | -0.08 – 1.04 | .092 | 0.08 | 0.33 | .01 | -0.56 – 0.72 | .813 |
| R² | 0.053 | | | | | 0.019 | | | | |

Note: Outgroup (0 = ingroup interaction, 1 = outgroup interaction) = effect of watching the video including an interaction with the outgroup compared to an interaction with the ingroup (Group Membership factor); Positive (0 = negative interaction, 1 = positive interaction) = effect of watching the video including positive contact compared to negative (Valence factor). NFC = Need for Closure, a measure of individuals' desire for certainty and aversion to ambiguity.

**Table S11. The moderation of NFC in the models for Cultural Deprovincialization, Group Deprovincialization, and Environmental concern, Study 1**

|  | ***Cultural Deprovincialization*** | | | | | ***Group Deprovincialization*** | | | | | ***Environmental concern*** | | | | |
| --- | --- | --- | --- | --- | --- | --- | --- | --- | --- | --- | --- | --- | --- | --- | --- |
|  | b | SE | β | 95% CI | p | b | SE | β | 95% CI | p | b | SE | β | 95% CI | p |
| Intercept | 7.19 | 0.46 | .00 | 6.28 – 8.10 | <.001 | 7.16 | 0.40 | .00 | 6.38 – 7.94 | <.001 | 6.32 | 0.46 | .01 | 5.42 – 7.22 | <.001 |
| Outgroup | -0.81 | 0.67 | -.02 | -2.12 – 0.50 | .225 | -0.80 | 0.57 | -.06 | -1.93 – 0.32 | .162 | 0.30 | 0.66 | .04 | -1.00 – 1.60 | .652 |
| Positive | -0.88 | 0.65 | .03 | -2.16 – 0.41 | .182 | -0.75 | 0.56 | .10 | -1.86 – 0.35 | .180 | -0.31 | 0.65 | -.00 | -1.59 – 0.96 | .629 |
| NFC | -0.27 | 0.11 | -.15 | -0.48 – -0.06 | .012 | -0.19 | 0.09 | -.06 | -0.37 – -0.01 | .036 | -0.05 | 0.11 | -.02 | -0.26 – 0.15 | .603 |
| Outgroup × Positive | 1.46 | 0.96 | .05 | -0.42 – 3.35 | .128 | 0.78 | 0.82 | .03 | -0.84 – 2.39 | .345 | -0.15 | 0.95 | -.07 | -2.02 – 1.71 | .872 |
| Outgroup × NFC | 0.16 | 0.15 | .01 | -0.14 – 0.46 | .288 | 0.15 | 0.13 | .04 | -0.11 – 0.41 | .250 | -0.02 | 0.15 | -.02 | -0.32 – 0.28 | .886 |
| Positive × NFC | 0.20 | 0.15 | .02 | -0.10 – 0.50 | .187 | 0.21 | 0.13 | .07 | -0.05 – 0.46 | .116 | 0.11 | 0.15 | .04 | -0.19 – 0.40 | .483 |
| Outgroup × Positive × NFC | -0.30 | 0.22 | -.07 | -0.74 – 0.13 | .174 | -0.16 | 0.19 | -.04 | -0.54 – 0.22 | .403 | -0.03 | 0.22 | -.01 | -0.46 – 0.40 | .894 |
| R² | 0.031 | | | | | 0.027 | | | | | 0.009 | | | | |

Note: Outgroup (0 = ingroup interaction, 1 = outgroup interaction) = effect of watching the video including an interaction with the outgroup compared to an interaction with the ingroup (Group Membership factor); Positive (0 = negative interaction, 1 = positive interaction) = effect of watching the video including positive contact compared to negative (Valence factor). NFC = Need for Closure, a measure of individuals' desire for certainty and aversion to ambiguity.

**Table S12. The moderation of NFC in the models for AUT dimensions, Study 1**

|  | ***Flexibility – AUT*** | | | | | ***Originality – AUT*** | | | | |
| --- | --- | --- | --- | --- | --- | --- | --- | --- | --- | --- |
|  | *b* | *SE* | β | *95% CI* | *p* | *b* | *SE* | β | *95% CI* | *p* |
| Intercept | 5.45 | 1.24 | -.00 | 3.01 – 7.88 | <.001 | 0.12 | 0.32 | .00 | -0.51 – 0.75 | .707 |
| Outgroup | 0.60 | 1.79 | -.07 | -2.91 – 4.11 | .737 | 0.65 | 0.46 | -.02 | -0.25 – 1.56 | .155 |
| Positive | 3.26 | 1.75 | .07 | -0.18 – 6.71 | .063 | 0.49 | 0.45 | -.00 | -0.39 – 1.38 | .274 |
| NFC | -0.20 | 0.28 | -.13 | -0.76 – 0.36 | .487 | 0.05 | 0.07 | -.05 | -0.10 – 0.19 | .515 |
| Outgroup× Positive | -3.65 | 2.56 | -.09 | -8.68 – 1.38 | .155 | -0.81 | 0.66 | -.07 | -2.10 – 0.48 | .219 |
| Outgroup × NFC | -0.11 | 0.41 | .04 | -0.92 – 0.69 | .781 | -0.14 | 0.11 | -.05 | -0.35 – 0.06 | .175 |
| Positive × NFC | -0.58 | 0.41 | -.04 | -1.38 – 0.22 | .155 | -0.10 | 0.11 | -.01 | -0.30 – 0.11 | .357 |
| Outgroup × Positive × NFC | 0.65 | 0.59 | .05 | -0.52 – 1.82 | .277 | 0.15 | 0.15 | .05 | -0.15 – 0.45 | .322 |
| R² | 0.043 | | | | | 0.012 | | | | |

Note: Outgroup (0 = ingroup interaction, 1 = outgroup interaction) = effect of watching the video including an interaction with the outgroup compared to an interaction with the ingroup (Group Membership factor); Positive (0 = negative interaction, 1 = positive interaction) = effect of watching the video including positive contact compared to negative (Valence factor). NFC = Need for Closure, a measure of individuals' desire for certainty and aversion to ambiguity.

**Table S13. The moderation of Curiosity in the models for MARS, and RAT, Study 1**

|  | ***MARS*** | | | | | ***RAT*** | | | | |
| --- | --- | --- | --- | --- | --- | --- | --- | --- | --- | --- |
|  | *b* | *SE* | β | *95% CI* | *p* | *b* | *SE* | β | *95% CI* | *p* |
| Intercept | 2.82 | 0.68 | .01 | 1.47 – 4.16 | <.001 | 1.80 | 0.77 | .00 | 0.30 – 3.31 | .019 |
| Outgroup | -0.85 | 0.95 | -.05 | -2.72 – 1.02 | .371 | -1.15 | 1.07 | .09 | -3.25 – 0.95 | .284 |
| Positive | -0.20 | 1.00 | -.03 | -2.17 – 1.77 | .843 | -1.73 | 1.13 | -.03 | -3.95 – 0.48 | .125 |
| Curiosity | 0.09 | 0.13 | .09 | -0.16 – 0.35 | .461 | -0.01 | 0.14 | .10 | -0.29 – 0.27 | .946 |
| Outgroup × Positive | 1.62 | 1.47 | -.07 | -1.26 – 4.50 | .269 | 2.51 | 1.65 | .05 | -0.72 – 5.75 | .128 |
| Outgroup × Curiosity | 0.17 | 0.18 | -.00 | -0.18 – 0.53 | .340 | 0.24 | 0.20 | .01 | -0.16 – 0.64 | .231 |
| Positive × Curiosity | 0.06 | 0.19 | -.05 | -0.31 – 0.43 | .745 | 0.29 | 0.21 | .02 | -0.13 – 0.70 | .172 |
| Outgroup × Positive × Curiosity | -0.38 | 0.27 | -.07 | -0.91 – 0.16 | .166 | -0.43 | 0.31 | -.07 | -1.03 – 0.17 | .163 |
| R² | 0.025 | | | | | 0.026 | | | | |

Note: Outgroup (0 = ingroup interaction, 1 = outgroup interaction) = effect of watching the video including an interaction with the outgroup compared to an interaction with the ingroup (Group Membership factor); Positive (0 = negative interaction, 1 = positive interaction) = effect of watching the video including positive contact compared to negative (Valence factor). Curiosity = a measure of individuals ‘desire to acquire knowledge and explore new ideas.

**Table S14. The moderation of Curiosity in the models for Cultural Deprovincialization, Group Deprovincialization, and Environmental concern, Study 1**

|  | ***Cultural Deprovincialization*** | | | | | ***Group Deprovincialization*** | | | | | ***Environmental concern*** | | | | |
| --- | --- | --- | --- | --- | --- | --- | --- | --- | --- | --- | --- | --- | --- | --- | --- |
|  | *b* | *SE* | β | *95% CI* | *p* | *b* | *SE* | β | *95% CI* | *p* | *b* | *SE* | β | *95% CI* | *p* |
| Intercept | 4.36 | 0.49 | .00 | 3.40 – 5.33 | <.001 | 5.45 | 0.44 | .01 | 4.59 – 6.31 | <.001 | 5.52 | 0.52 | .01 | 4.51 – 6.54 | <.001 |
| Outgroup | -1.02 | 0.68 | -.01 | -2.37 – 0.32 | .137 | -0.93 | 0.61 | -.05 | -2.13 – 0.27 | .128 | -0.28 | 0.72 | .05 | -1.70 – 1.13 | .694 |
| Positive | -0.02 | 0.72 | -.01 | -1.44 – 1.39 | .973 | 0.72 | 0.64 | .06 | -0.54 – 1.99 | .263 | 0.45 | 0.76 | -.02 | -1.04 – 1.94 | .553 |
| Curiosity | 0.32 | 0.09 | .39 | 0.14 – 0.50 | .001 | 0.17 | 0.08 | .24 | 0.01 – 0.33 | .40 | 0.11 | 0.10 | .11 | -0.08 – 0.30 | .268 |
| Outgroup × Positive | 0.41 | 1.06 | .01 | -1.66 – 2.49 | .695 | -0.35 | 0.94 | -.00 | -2.20 – 1.50 | .712 | 0.11 | 1.11 | -.09 | -2.08 – 2.29 | .924 |
| Outgroup × Curiosity | 0.19 | 0.13 | .07 | -0.07 – 0.44 | .148 | 0.16 | 0.12 | .11 | -0.07 – 0.39 | .173 | 0.10 | 0.14 | .03 | -0.17 – 0.37 | .468 |
| Positive × Curiosity | -0.00 | 0.13 | -.02 | -0.27 – 0.26 | .983 | -0.12 | 0.12 | -.05 | -0.35 – 0.12 | .327 | -0.06 | 0.14 | -.05 | -0.34 – 0.22 | .664 |
| Outgroup × Positive × Curiosity | -0.07 | 0.20 | -.02 | -0.46 – 0.31 | .708 | 0.07 | 0.18 | .02 | -0.28 – 0.41 | .704 | -0.08 | 0.21 | -.02 | -0.49 – 0.32 | .696 |
| R² | 0.155 | | | | | 0.083 | | | | | 0.023 | | | | |

Note: Outgroup (0 = ingroup interaction, 1 = outgroup interaction) = effect of watching the video including an interaction with the outgroup compared to an interaction with the ingroup (Group Membership factor); Positive (0 = negative interaction, 1 = positive interaction) = effect of watching the video including positive contact compared to negative (Valence factor). Curiosity = a measure of individuals ‘desire to acquire knowledge and explore new ideas.

**Table S15. The moderation of Curiosity in the models for AUT dimensions, Study 1**

|  | ***Fluency – AUT*** | | | | | ***Flexibility – AUT*** | | | | |
| --- | --- | --- | --- | --- | --- | --- | --- | --- | --- | --- |
|  | *b* | *SE* | β | *95% CI* | *p* | *b* | *SE* | β | *95% CI* | *p* |
| Intercept | 6.38 | 2.26 | .01 | 1.93 – 10.82 | .005 | 6.38 | 2.26 | .01 | 1.93 – 10.82 | .005 |
| Outgroup | -4.03 | 3.15 | -.06 | -10.23 – 2.16 | .202 | -4.03 | 3.15 | -.07 | -10.23 – 2.16 | .202 |
| Positive | -1.83 | 3.32 | .03 | -8.36 – 4.70 | .582 | -1.83 | 3.32 | .07 | -8.36 – 4.70 | .582 |
| Curiosity | 0.23 | 0.42 | .13 | -0.60 – 1.06 | .586 | 0.23 | 0.42 | .08 | -0.60 – 1.06 | .586 |
| Outgroup × Positive | 6.05 | 4.85 | -.09 | -3.49 – 15.59 | .213 | 6.05 | 4.85 | -.10 | -3.49 – 15.59 | .213 |
| Outgroup × Curiosity | 0.83 | 0.60 | .02 | -0.35 – 2.01 | .169 | 0.83 | 0.60 | .01 | -0.35 – 2.01 | .169 |
| Positive × Curiosity | 0.52 | 0.62 | -.02 | -0.69 – 1.73 | .398 | 0.52 | 0.62 | .01 | -0.69 – 1.73 | .398 |
| Outgroup × Positive × Curiosity | -1.43 | 0.90 | -.08 | -3.20 – 0.34 | .114 | -1.43 | 0.90 | -.05 | -3.20 – 0.34 | .114 |
| R² | 0.035 | | | | | 0.030 | | | | |

Note: Outgroup (0 = ingroup interaction, 1 = outgroup interaction) = effect of watching the video including an interaction with the outgroup compared to an interaction with the ingroup (Group Membership factor); Positive (0 = negative interaction, 1 = positive interaction) = effect of watching the video including positive contact compared to negative (Valence factor). Curiosity = a measure of individuals ‘desire to acquire knowledge and explore new ideas.

**Table S15 (continued). The moderation of Curiosity in the models for AUT dimensions, Study 1**

|  | ***Elaboration – AUT*** | | | | | ***Originality – AUT*** | | | | |
| --- | --- | --- | --- | --- | --- | --- | --- | --- | --- | --- |
|  | *b* | *SE* | β | *95% CI* | *p* | *b* | *SE* | β | *95% CI* | *p* |
| Intercept | 6.39 | 2.89 | .01 | 0.70 – 12.07 | .028 | 0.12 | 0.36 | .00 | -0.59 – 0.83 | .743 |
| Outgroup | -2.63 | 4.03 | -.05 | -10.56 – 5.30 | .514 | -0.10 | 0.50 | -.02 | -1.09 – 0.89 | .838 |
| Positive | -3.02 | 4.25 | .03 | -11.38 – 5.34 | .478 | -0.16 | 0.53 | -.01 | -1.20 – 0.88 | .763 |
| Curiosity | 0.50 | 0.54 | .14 | -0.57 – 1.57 | .358 | 0.04 | 0.07 | .09 | -0.09 – 0.17 | .564 |
| Outgroup × Positive | 6.58 | 6.21 | -.05 | -5.63 – 18.79 | .290 | 0.10 | 0.78 | -.07 | -1.42 – 1.63 | .893 |
| Outgroup × Curiosity | 0.52 | 0.77 | -.02 | -0.99 – 2.03 | .495 | 0.03 | 0.10 | .00 | -0.16 – 0.22 | .754 |
| Positive × Curiosity | 0.74 | 0.79 | -.00 | -0.81 – 2.29 | .347 | 0.04 | 0.10 | .01 | -0.15 – 0.24 | .652 |
| Outgroup × Positive × Curiosity | -1.47 | 1.15 | -.07 | -3.73 – 0.80 | .204 | -0.05 | 0.14 | -.02 | -0.34 – 0.23 | .709 |
| R² | 0.031 | | | | | 0.013 | | | | |

Note: Outgroup (0 = ingroup interaction, 1 = outgroup interaction) = effect of watching the video including an interaction with the outgroup compared to an interaction with the ingroup (Group Membership factor); Positive (0 = negative interaction, 1 = positive interaction) = effect of watching the video including positive contact compared to negative (Valence factor). Curiosity = a measure of individuals ‘desire to acquire knowledge and explore new ideas.

**Table S16. Means, standard deviations, and intercorrelations among variable in Study 2**

|  | *M* | *SD* | 1. | 2. | 3. | 4. | 5. | 6. | 7. | 8. | 9. | 10. | 11. | 12. | 13. | 14. |
| --- | --- | --- | --- | --- | --- | --- | --- | --- | --- | --- | --- | --- | --- | --- | --- | --- |
| 1.Cognitive Flexibility | 4.92 | 0.86 |  |  |  |  |  |  |  |  |  |  |  |  |  |  |
| 2.Fluency – AUT | 7.83 | 3.92 | .12^**^ |  |  |  |  |  |  |  |  |  |  |  |  |  |
| 3.Elaboration – AUT | 9.06 | 4.78 | .11^**^ | .89^***^ |  |  |  |  |  |  |  |  |  |  |  |  |
| 4.Flexibility – AUT | 5.01 | 2.59 | .09^*^ | .80^***^ | .78^***^ |  |  |  |  |  |  |  |  |  |  |  |
| 5.Originality – AUT | 0.40 | 0.69 | .07 | .43^***^ | .54^***^ | .58^***^ |  |  |  |  |  |  |  |  |  |  |
| 6.MARS | 3.47 | 1.25 | -.05 | .01 | .01 | -.03 | -.04 |  |  |  |  |  |  |  |  |  |
| 7.RAT | 1.94 | 1.49 | -.00 | .07 | .03 | .07 | -.00 | .08 |  |  |  |  |  |  |  |  |
| 8.Cultural Deprovincialization | 5.88 | 0.96 | .16^***^ | .07 | .08 | .03 | .05 | .02 | .08 |  |  |  |  |  |  |  |
| 9.Group Deprovincialization | 6.25 | 0.86 | .13^**^ | .14^***^ | .13^**^ | .11^**^ | .06 | .08 | .13^**^ | .50^***^ |  |  |  |  |  |  |
| 10.Environmental Concern | 6.10 | 0.88 | -.01 | .05 | .06 | .05 | .09^*^ | .06 | .08 | .22^***^ | .29^***^ |  |  |  |  |  |
| 11.SDO | 2.05 | 0.98 | -.06 | -.08 | -.07 | -.01 | .00 | -.01 | -.08 | -.41^***^ | -.34^***^ | -.29^***^ |  |  |  |  |
| 12.NFC | 4.19 | 0.83 | -.25^***^ | -.07 | -.09^*^ | -.09^*^ | -.06 | .06 | -.02 | -.19^***^ | -.17^***^ | -.08 | .13^**^ |  |  |  |
| 13.Curiosity | 5.18 | 0.93 | .37^***^ | .12^**^ | .11^*^ | .11^*^ | .07 | -.02 | .14^**^ | .37^***^ | .32^***^ | .10^*^ | -.16^***^ | -.32^***^ |  |  |
| 14.Positive direct contact | 2.73 | 1.07 | .10^*^ | .06 | .11^**^ | .09^*^ | .10^*^ | -.01 | .02 | .22^***^ | .14^**^ | .05 | -.10^*^ | -.13^**^ | .20^***^ |  |
| 15.Negative direct contact | 1.48 | 0.68 | .05 | .03 | .04 | .06 | .06 | .02 | -.04 | -.12^**^ | -.11^**^ | -.04 | .18^***^ | -.01 | .06 | .30^***^ |

*Note.* ^*^ *p <* .05, ^**^ *p <* .01, ^***^ *p <* .001.

**Table S17**. **Manipulation Check Results of Study 2**

| **Manipulation Check** | ***F*** | ***df*** | ***p*** | ***ηp²*** |
| --- | --- | --- | --- | --- |
|  |  |  |  |  |
| Awareness of being in a different group than Sana — Condition differences | 1.87 | 3, 569 | .314 | .01 |
| Perceived typicality/representativeness of Sana — Semantic distance main effect | 22.35 | 1, 570 | < .001 | .04 |
| Perceived typicality/representativeness of Sana — Valence main effect | 4.52 | 1, 570 | .034 | .01 |
| Perceived positive valence of video — Valence main effect | 612.05 | 1, 570 | < .001 | .52 |
| Perceived positive valence of video — Semantic distance × Valence interaction | 9.63 | 1, 570 | .002 | .02 |
| Perceived negative valence of video — Valence main effect | 473.51 | 1, 570 | < .001 | .45 |
| Positive emotions experienced — Valence main effect | 347.43 | 1, 570 | < .001 | .38 |
| Positive emotions — Semantic distance × Valence interaction | 9.85 | 1, 570 | .002 | .02 |
| Negative emotions experienced — Valence main effect | 396.13 | 1, 570 | < .001 | .41 |
| Negative emotions — Semantic distance main effect | 14.96 | 1, 570 | < .001 | .03 |
| Plausibility of video — Condition differences | 0.911 | 3, 570 | .435 | .00 |
| Difficulty understanding video — Semantic distance main effect | 26.66 | 1, 570 | < .001 | .04 |

*Note.* Semantic distance = atypical vs. prototypical outgroup member; Valence = positive vs. negative interaction. Unless otherwise indicated, tests are ANOVAs testing condition differences or effects of Semantic distance and Valence.

**Table S18.** **The effects of the manipulations (Semantic Distance and Valence) on MARS, RAT, and Environmental Concern, Study 2**

|  | ***MARS*** | | | | | ***RAT*** | | | | | ***Environmental Concern*** | | | | |
| --- | --- | --- | --- | --- | --- | --- | --- | --- | --- | --- | --- | --- | --- | --- | --- |
|  | *b* | *SE* | β | *95% CI* | *𝑝* | *b* | *SE* | β | *95% CI* | *𝑝* | *b* | *SE* | β | *95% CI* | *𝑝* |
| Intercept | 3.60 | 0.11 | .00 | 3.39 – 3.82 | <.001 | 1.99 | 0.13 | -.00 | 1.74 – 2.25 | <.001 | 6.17 | 0.08 | .00 | 6.02 – 6.32 | <.001 |
| Atypical | -0.14 | 0.15 | -.04 | -0.43 – 0.16 | .356 | -0.20 | 0.18 | -.09 | -0.55 – 0.14 | .250 | -0.12 | 0.11 | -.05 | -0.32 – 0.09 | .270 |
| Positive | -0.16 | 0.15 | -.05 | -0.46 – 0.13 | .279 | 0.14 | 0.18 | .03 | -0.21 – 0.50 | .419 | -0.05 | 0.11 | -.01 | -0.26 – 0.16 | .631 |
| Atypical × Positive | 0.09 | 0.21 | .02 | -0.32 – 0.50 | .673 | -0.10 | 0.25 | -.02 | -0.59 – 0.39 | .691 | 0.07 | 0.15 | .02 | -0.22 – 0.36 | .640 |
| R^2^ | 0.004 | | | | | 0.009 | | | | | 0.003 | | | | |

Note. Atypical (0 = typical interaction, 1 = atypical interaction) = effect of atypical outgroup member compared to prototypical (Semantic distance factor); Positive (0 = negative interaction, 1 = positive interaction) = effect of positive intergroup contact compared to negative (Valence factor).

**Table S19. The effects of the manipulations (Semantic Distance and Valence) on Cognitive Flexibility (CF) items, Study 2**

|  | ***CF_1*** | | | | | ***CF_2*** | | | | |
| --- | --- | --- | --- | --- | --- | --- | --- | --- | --- | --- |
|  | *b* | *SE* | β | *95% CI* | *𝑝* | *b* | *SE* | β | 95% CI | 𝑝 |
| Intercept | 4.92 | 0.11 | -.00 | 4.71 – 5.14 | <.001 | 4.06 | 0.21 | -.00 | 3.65 – 4.47 | <.001 |
| Atypical | 0.06 | 0.15 | -.03 | -0.24 – 0.36 | .683 | 0.09 | 0.29 | -.01 | -0.47 – 0.65 | .745 |
| Positive | -0.01 | 0.15 | -.06 | -0.31 – 0.28 | .927 | 0.06 | 0.29 | -.02 | -0.50 – 0.63 | .826 |
| Atypical × Positive | -0.28 | 0.21 | -.05 | -0.69 – 0.14 | .195 | -0.32 | 0.40 | -.03 | -1.11 – 0.47 | .425 |
| R^2^ | 0.007 | | | | | 0.002 | | | | |

Note. Atypical (0 = typical interaction, 1 = atypical interaction) = effect of atypical outgroup member compared to prototypical (Semantic distance factor); Positive (0 = negative interaction, 1 = positive interaction) = effect of positive intergroup contact compared to negative (Valence factor).

**Table S19 (continued). The effects of the manipulations (Semantic Distance and Valence) on Cognitive Flexibility (CF) items, Study 2**

|  | ***CF_3*** | | | | | ***CF_4*** | | | | |
| --- | --- | --- | --- | --- | --- | --- | --- | --- | --- | --- |
|  | *b* | *SE* | β | *95% CI* | *𝑝* | *b* | *SE* | β | *95% CI* | *𝑝* |
| Intercept | 4.32 | 0.14 | -.00 | 4.04 – 4.60 | <.001 | 4.73 | 0.11 | -.00 | 4.51 – 4.95 | <.001 |
| Atypical | 0.01 | 0.20 | -.00 | -0.37 – 0.39 | .959 | -0.01 | 0.15 | -.03 | -0.31 – 0.29 | .952 |
| Positive | -0.02 | 0.20 | -.01 | -0.40 – 0.37 | .939 | 0.17 | 0.15 | .04 | -0.13 – 0.48 | .260 |
| Atypical × Positive | -0.04 | 0.27 | -.01 | -0.58 – 0.50 | .883 | -0.16 | 0.22 | -.03 | -0.58 – 0.27 | .463 |
| R^2^ | 0.000 | | | | | 0.004 | | | | |

Note. Atypical (0 = typical interaction, 1 = atypical interaction) = effect of atypical outgroup member compared to prototypical (Semantic distance factor); Positive (0 = negative interaction, 1 = positive interaction) = effect of positive intergroup contact compared to negative (Valence factor).

**Table S20. The effects of the manipulations (Semantic Distance and Valence) on AUT dimensions, Study 2**

|  | ***Fluency – AUT*** | | | | | ***Elaboration – AUT*** | | | | | ***Originality – AUT*** | | | | |
| --- | --- | --- | --- | --- | --- | --- | --- | --- | --- | --- | --- | --- | --- | --- | --- |
|  | *b* | *SE* | β | *95% CI* | *𝑝* | *b* | *SE* | β | *95% CI* | *𝑝* | *b* | *SE* | β | *95% CI* | *𝑝* |
| Intercept | 8.07 | 0.34 | -.00 | 7.40 – 8.74 | <.001 | 9.28 | 0.42 | -.00 | 8.46 – 10.10 | <.001 | 0.41 | 0.06 | -.00 | 0.29 – 0.52 | <.001 |
| Atypical | -0.33 | 0.47 | -.05 | -1.25 – 0.58 | .476 | -0.26 | 0.57 | -.05 | -1.38 – 0.86 | .648 | 0.00 | 0.08 | -.03 | -0.16 – 0.16 | .994 |
| Positive | -0.07 | 0.47 | -.02 | -1.00 – 0.85 | .874 | 0.08 | 0.57 | -.02 | -1.05 – 1.21 | .886 | 0.03 | 0.08 | -.01 | -0.13 – 0.19 | .705 |
| Atypical × Positive | -0.11 | 0.66 | -.01 | -1.40 – 1.18 | .864 | -0.51 | 0.80 | -.03 | -2.08 – 1.07 | .528 | -0.10 | 0.12 | -.03 | -0.32 – 0.13 | .410 |
| R^2^ | 0.003 | | | | | 0.004 | | | | | 0.003 | | | | |

Note. AUT = Alternative Uses Task (creativity). Atypical (0 = typical interaction, 1 = atypical interaction) = effect of atypical outgroup member compared to prototypical (Semantic distance factor); Positive (0 = negative interaction, 1 = positive interaction) = effect of positive intergroup contact compared to negative (Valence factor).

**Table S21. The moderation of SDO in the models for MARS and RAT, Study 2**

|  | ***MARS*** | | | | | ***RAT*** | | | | |
| --- | --- | --- | --- | --- | --- | --- | --- | --- | --- | --- |
|  | *b* | *SE* | β | *95% CI* | *𝑝* | *b* | *SE* | β | *95% CI* | *𝑝* |
| Intercept | 3.54 | 0.26 | .00 | 3.03 – 4.05 | <.001 | 2.16 | 0.31 | -.00 | 1.56 – 2.76 | <.001 |
| Atypical | 0.01 | 0.36 | -.04 | -0.69 – 0.71 | .969 | -0.22 | 0.42 | -.08 | -1.05 – 0.60 | .594 |
| Positive | 0.03 | 0.36 | -.05 | -0.68 – 0.73 | .943 | 0.56 | 0.42 | .03 | -0.27 – 1.39 | .188 |
| SDO | 0.03 | 0.12 | -.01 | -0.21 – 0.27 | .804 | -0.09 | 0.15 | -.08 | -0.38 – 0.19 | .527 |
| Atypical × Positive | -0.27 | 0.50 | .02 | -1.25 – 0.70 | .581 | -0.62 | 0.59 | -.02 | -1.77 – 0.54 | .294 |
| Atypical × SDO | -0.07 | 0.16 | .01 | -0.39 – 0.24 | .646 | 0.02 | 0.19 | .05 | -0.35 – 0.39 | .907 |
| Positive × SDO | -0.10 | 0.17 | -.00 | -0.42 – 0.23 | .570 | -0.20 | 0.20 | -.02 | -0.59 – 0.19 | .312 |
| Atypical × Positive × SDO | 0.18 | 0.22 | .03 | -0.26 – 0.61 | .424 | 0.25 | 0.26 | .04 | -0.27 – 0.77 | .341 |
| R^2^ | 0.005 | | | | | 0.019 | | | | |

Note. Atypical (0 = typical interaction, 1 = atypical interaction) = effect of atypical outgroup member compared to prototypical (Semantic distance factor). Positive (0 = negative interaction, 1 = positive interaction) = effect of positive intergroup contact compared to negative (Valence factor). SDO = Social Dominance Orientation, a measure of preference for hierarchical versus egalitarian social relations.

**Table S22. The moderation of SDO in the models for Group Deprovincialization and Environmental concern, Study 2**

|  | ***Group Deprovincialization*** | | | | | ***Environmental concern*** | | | | |
| --- | --- | --- | --- | --- | --- | --- | --- | --- | --- | --- |
|  | *b* | *SE* | β | *95% CI* | *𝑝* | *b* | *SE* | β | *95% CI* | *𝑝* |
| Intercept | 6.89 | 0.17 | .00 | 6.56 – 7.22 | <.001 | 6.53 | 0.17 | -.00 | 6.19 – 6.87 | <.001 |
| Atypical | 0.15 | 0.23 | -.09 | -0.30 – 0.60 | .500 | 0.01 | 0.24 | -.01 | -0.46 – 0.48 | .973 |
| Positive | 0.06 | 0.23 | -.02 | -0.39 – 0.51 | .803 | 0.35 | 0.24 | -.02 | -0.12 – 0.82 | .148 |
| SDO | -0.28 | 0.08 | -.32 | -0.44 – -0.13 | <.001 | -0.19 | 0.08 | -.29 | -0.36 – -0.03 | .020 |
| Atypical × Positive | -0.60 | 0.32 | -.03 | -1.23 – 0.02 | .060 | -0.28 | 0.33 | .00 | -0.94 – 0.38 | .405 |
| Atypical × SDO | -0.12 | 0.10 | -.00 | -0.32 – 0.08 | .228 | -0.02 | 0.11 | .03 | -0.23 – 0.19 | .847 |
| Positive × SDO | -0.01 | 0.11 | .06 | -0.23 – 0.20 | .896 | -0.19 | 0.11 | -.06 | -0.41 – 0.03 | .093 |
| Atypical × Positive × SDO | 0.24 | 0.14 | .07 | -0.04 – 0.52 | .093 | 0.14 | 0.15 | .04 | -0.15 – 0.44 | .335 |
| R^2^ | 0.133 | | | | | 0.090 | | | | |

Note. Atypical (0 = typical interaction, 1 = atypical interaction) = effect of atypical outgroup member compared to prototypical (Semantic distance factor). Positive (0 = negative interaction, 1 = positive interaction) = effect of positive intergroup contact compared to negative (Valence factor). SDO = Social Dominance Orientation, a measure of preference for hierarchical versus egalitarian social relations.

|  | ***Fluency - AUT*** | | | | | ***Elaboration - AUT*** | | | | |
| --- | --- | --- | --- | --- | --- | --- | --- | --- | --- | --- |
|  | *b* | *SE* | β | *95% CI* | *𝑝* | *b* | *SE* | β | *95% CI* | *𝑝* |
| Intercept | 8.54 | 0.81 | .00 | 6.96 – 10.13 | <.001 | 10.04 | 0.99 | -.00 | 8.09 – 11.99 | <.001 |
| Atypical | -0.08 | 1.11 | -.04 | -2.25 – 2.10 | .945 | 0.04 | 1.36 | -.05 | -2.63 – 2.71 | .976 |
| Positive | -0.07 | 1.11 | -.02 | -2.26 – 2.12 | .950 | -0.16 | 1.36 | -.02 | -2.84 – 2.52 | .904 |
| SDO | -0.23 | 0.39 | -.08 | -0.99 – 0.52 | .545 | -0.38 | 0.47 | -.06 | -1.30 – 0.55 | .426 |
| Atypical × Positive | 0.07 | 1.55 | -.01 | -2.97 – 3.11 | .963 | -1.03 | 1.90 | -.03 | -4.76 – 2.69 | .587 |
| Atypical × SDO | -0.10 | 0.49 | -.02 | -1.07 – 0.88 | .845 | -0.10 | 0.61 | .00 | -1.29 – 1.09 | .871 |
| Positive × SDO | -0.01 | 0.52 | -.01 | -1.03 – 1.01 | .988 | 0.12 | 0.64 | .02 | -1.13 – 1.37 | .852 |
| Atypical × Positive × SDO | -0.11 | 0.69 | -.01 | -1.47 – 1.25 | .878 | 0.22 | 0.85 | .01 | -1.45 – 1.88 | .797 |
| R^2^ | 0.010 | | | | | 0.009 | | | | |

**Table S23. The moderation of SDO in the models for AUT dimensions, Study 2**

Note. Atypical (0 = typical interaction, 1 = atypical interaction) = effect of atypical outgroup member compared to prototypical (Semantic distance factor). Positive (0 = negative interaction, 1 = positive interaction) = effect of positive intergroup contact compared to negative (Valence factor). SDO = Social Dominance Orientation, a measure of preference for hierarchical versus egalitarian social relations.

**Table S23 (continued). The moderation of SDO in the models for AUT dimensions, Study 2**

|  | ***Flexibility - AUT*** | | | | | ***Originality - AUT*** | | | | |
| --- | --- | --- | --- | --- | --- | --- | --- | --- | --- | --- |
|  | *b* | *SE* | β | *95% CI* | *𝑝* | *b* | *SE* | β | *95% CI* | *𝑝* |
| Intercept | 5.60 | 0.53 | .00 | 4.56 – 6.65 | <.001 | 0.48 | 0.14 | -.00 | 0.20 – 0.76 | .001 |
| Atypical | -0.74 | 0.73 | -.09 | -2.18 – 0.70 | .313 | -0.01 | 0.20 | -.03 | -0.39 – 0.38 | .978 |
| Positive | -0.72 | 0.73 | -.04 | -2.17 – 0.72 | .326 | -0.05 | 0.20 | -.01 | -0.43 – 0.34 | .814 |
| SDO | -0.08 | 0.25 | -.01 | -0.58 – 0.42 | .761 | -0.04 | 0.07 | -.00 | -0.17 – 0.09 | .566 |
| Atypical × Positive | 0.80 | 1.02 | .03 | -1.21 – 2.81 | .434 | -0.21 | 0.27 | -.04 | -0.75 – 0.33 | .443 |
| Atypical × SDO | 0.05 | 0.33 | -.01 | -0.59 – 0.69 | .873 | 0.01 | 0.09 | .02 | -0.17 – 0.18 | .954 |
| Positive × SDO | 0.16 | 0.34 | .01 | -0.52 – 0.83 | .644 | 0.04 | 0.09 | .05 | -0.14 – 0.22 | .669 |
| Atypical × Positive × SDO | -0.22 | 0.46 | -.02 | -1.12 – 0.68 | .631 | 0.05 | 0.12 | .02 | -0.19 – 0.29 | .663 |
| R^2^ | 0.011 | | | | | 0.006 | | | | |

Note. Atypical (0 = typical interaction, 1 = atypical interaction) = effect of atypical outgroup member compared to prototypical (Semantic distance factor). Positive (0 = negative interaction, 1 = positive interaction) = effect of positive intergroup contact compared to negative (Valence factor). SDO = Social Dominance Orientation, a measure of preference for hierarchical versus egalitarian social relations.

**Table S24. The moderation of NFC in the models for MARS and RAT, Study 2**

|  | ***MARS*** | | | | | ***RAT*** | | | | |
| --- | --- | --- | --- | --- | --- | --- | --- | --- | --- | --- |
|  | *b* | *SE* | β | *95% CI* | *𝑝* | *b* | *SE* | β | *95% CI* | *𝑝* |
| Intercept | 3.08 | 0.59 | .00 | 1.93 – 4.23 | <.001 | 2.76 | 0.70 | -.00 | 1.38 – 4.13 | <.001 |
| Atypical | -0.44 | 0.78 | -.04 | -1.97 – 1.10 | .578 | -1.07 | 0.93 | -.08 | -2.90 – 0.76 | .252 |
| Positive | 0.55 | 0.80 | -.05 | -1.03 – 2.12 | .495 | -0.73 | 0.95 | .03 | -2.60 – 1.15 | .447 |
| NFC | 0.13 | 0.14 | .06 | -0.15 – 0.40 | .366 | -0.18 | 0.17 | -.03 | -0.51 – 0.14 | .267 |
| Atypical × Positive | -0.23 | 1.08 | .02 | -2.36 – 1.89 | .829 | 1.10 | 1.29 | -.02 | -1.43 – 3.64 | .393 |
| Atypical × NFC | 0.07 | 0.18 | .04 | -0.29 – 0.43 | .707 | 0.21 | 0.22 | .02 | -0.22 – 0.64 | .343 |
| Positive × NFC | -0.17 | 0.19 | -.04 | -0.54 – 0.20 | .365 | 0.21 | 0.22 | .02 | -0.23 – 0.65 | .350 |
| Atypical × Positive × NFC | 0.08 | 0.25 | .01 | -0.42 – 0.58 | .757 | -0.29 | 0.30 | -.04 | -0.88 – 0.30 | .340 |
| R^2^ | 0.011 | | | | | 0.011 | | | | |

Note. NFC = Need for Closure, a measure of individuals' desire for certainty and aversion to ambiguity. Atypical (0 = typical interaction, 1 = atypical interaction) = effect of atypical outgroup member compared to prototypical (Semantic distance factor). Positive (0 = negative interaction, 1 = positive interaction) = effect of positive intergroup contact compared to negative (Valence factor).

**Table S25. The moderation of NFC in the models for Cultural Deprovincialization, Group Deprovincialization, and Environmental concern, Study 2**

|  | ***Cultural Deprovincialization*** | | | | | ***Group Deprovincialization*** | | | | | ***Environmental concern*** | | | | |
| --- | --- | --- | --- | --- | --- | --- | --- | --- | --- | --- | --- | --- | --- | --- | --- |
|  | *b* | *SE* | β | *95% CI* | *𝑝* | *b* | *SE* | β | *95% CI* | *𝑝* | *b* | *SE* | β | *95% CI* | *𝑝* |
| Intercept | 6.86 | 0.44 | .00 | 5.99 – 7.73 | <.001 | 7.04 | 0.40 | -.00 | 6.26 – 7.82 | <.001 | 6.54 | 0.41 | .00 | 5.73 – 7.35 | <.001 |
| Atypical | 0.20 | 0.59 | -.05 | -0.96 – 1.36 | .732 | 0.16 | 0.53 | -.13 | -0.88 – 1.20 | .762 | 0.66 | 0.55 | -.04 | -0.42 – 1.74 | .231 |
| Positive | -0.37 | 0.60 | .02 | -1.56 – 0.81 | .537 | -0.12 | 0.54 | -.00 | -1.18 – 0.95 | .827 | -0.32 | 0.56 | -.01 | -1.42 – 0.78 | .572 |
| NFC | -0.20 | 0.10 | -.17 | -0.41 – 0.00 | .051 | -0.16 | 0.09 | -.17 | -0.35 – 0.02 | .082 | -0.09 | 0.10 | -.08 | -0.28 – 0.10 | .357 |
| Atypical × Positive | -0.21 | 0.81 | .08 | -1.81 – 1.39 | .799 | -0.33 | 0.73 | -.01 | -1.77 – 1.11 | .655 | -1.07 | 0.76 | .02 | -2.56 – 0.42 | .158 |
| Atypical × NFC | -0.11 | 0.14 | -.02 | -0.38 – 0.16 | .420 | -0.09 | 0.12 | -.02 | -0.33 – 0.16 | .496 | -0.18 | 0.13 | -.02 | -0.44 – 0.07 | .157 |
| Positive × NFC | 0.06 | 0.14 | .05 | -0.22 – 0.34 | .673 | 0.03 | 0.13 | .03 | -0.22 – 0.28 | .804 | 0.06 | 0.13 | .10 | -0.19 – 0.32 | .623 |
| Atypical × Positive × NFC | 0.13 | 0.19 | .03 | -0.25 – 0.50 | .507 | 0.07 | 0.17 | .02 | -0.27 – 0.41 | .691 | 0.27 | 0.18 | .06 | -0.08 – 0.62 | .128 |
| R^2^ | 0.046 | | | | | 0.047 | | | | | 0.023 | | | | |

Note. NFC = Need for Closure, a measure of individuals' desire for certainty and aversion to ambiguity. Atypical (0 = typical interaction, 1 = atypical interaction) = effect of atypical outgroup member compared to prototypical (Semantic distance factor). Positive (0 = negative interaction, 1 = positive interaction) = effect of positive intergroup contact compared to negative (Valence factor).

**Table S26. The moderation of NFC in the models for AUT dimensions, Study 2**

|  | ***Fluency - AUT*** | | | | | ***Elaboration - AUT*** | | | | |
| --- | --- | --- | --- | --- | --- | --- | --- | --- | --- | --- |
|  | *b* | *SE* | β | *95% CI* | *𝑝* | *b* | *SE* | β | *95% CI* | *𝑝* |
| Intercept | 10.42 | 1.83 | -.00 | 6.82 – 14.02 | <.001 | 12.29 | 2.24 | -.00 | 7.90 – 16.69 | <.001 |
| Atypical | -1.47 | 2.45 | -.05 | -6.27 – 3.34 | .549 | -0.67 | 2.98 | -.05 | -6.53 – 5.20 | .823 |
| Positive | 0.06 | 2.50 | -.01 | -4.85 – 4.97 | .981 | 0.29 | 3.05 | -.02 | -5.71 – 6.28 | .925 |
| NFC | -0.57 | 0.43 | -.08 | -1.42 – 0.29 | .192 | -0.73 | 0.53 | -.10 | -1.77 – 0.32 | .171 |
| Atypical × Positive | -1.12 | 3.38 | -.01 | -7.76 – 5.52 | .740 | -2.47 | 4.12 | -.03 | -10.57 – 5.62 | .549 |
| Atypical × NFC | 0.28 | 0.58 | .04 | -0.85 – 1.41 | .630 | 0.11 | 0.70 | .03 | -1.27 – 1.49 | .879 |
| Positive × NFC | -0.02 | 0.59 | .01 | -1.17 – 1.13 | .972 | -0.03 | 0.72 | .02 | -1.44 – 1.37 | .962 |
| Atypical × Positive × NFC | 0.23 | 0.79 | .01 | -1.33 – 1.78 | .772 | 0.45 | 0.97 | .02 | -1.44 – 2.35 | .639 |
| R^2^ | 0.011 | | | | | 0.015 | | | | |

Note. NFC = Need for Closure, a measure of individuals' desire for certainty and aversion to ambiguity. Atypical (0 = typical interaction, 1 = atypical interaction) = effect of atypical outgroup member compared to prototypical (Semantic distance factor). Positive (0 = negative interaction, 1 = positive interaction) = effect of positive intergroup contact compared to negative (Valence factor).

**Table S26 continued. The moderation of NFC in the models for AUT dimensions, Study 2**

|  | ***Flexibility - AUT*** | | | | | ***Originality - AUT*** | | | | |
| --- | --- | --- | --- | --- | --- | --- | --- | --- | --- | --- |
|  | *b* | *SE* | β | *95% CI* | *𝑝* | *b* | *SE* | β | *95% CI* | *𝑝* |
| Intercept | 6.92 | 1.21 | .00 | 4.54 – 9.29 | <.001 | 0.60 | 0.32 | -.00 | -0.03 – 1.24 | .064 |
| Atypical | -1.70 | 1.61 | -.09 | -4.86 – 1.46 | .292 | -0.21 | 0.43 | -.04 | -1.06 – 0.64 | .627 |
| Positive | -0.28 | 1.65 | -.04 | -3.51 – 2.96 | .867 | 0.31 | 0.44 | -.01 | -0.55 – 1.18 | .480 |
| NFC | -0.36 | 0.29 | -.08 | -0.92 – 0.21 | .213 | -0.05 | 0.08 | -.06 | -0.20 – 0.10 | .540 |
| Atypical × Positive | 0.80 | 2.23 | .03 | -3.57 – 5.17 | .719 | -0.24 | 0.60 | -.04 | -1.41 – 0.93 | .690 |
| Atypical × NFC | 0.26 | 0.38 | .03 | -0.48 – 1.01 | .489 | 0.05 | 0.10 | .04 | -0.15 – 0.25 | .617 |
| Positive × NFC | -0.02 | 0.39 | -.01 | -0.78 – 0.74 | .956 | -0.07 | 0.10 | -.03 | -0.27 – 0.14 | .527 |
| Atypical × Positive × NFC | -0.12 | 0.52 | -.01 | -1.14 – 0.91 | .821 | 0.03 | 0.14 | .01 | -0.24 – 0.31 | .816 |
| R^2^ | 0.018 | | | | | 0.008 | | | | |

Note. NFC = Need for Closure, a measure of individuals’ desire for certainty and aversion to ambiguity. Atypical (0 = typical interaction, 1 = atypical interaction) = effect of atypical outgroup member compared to prototypical (Semantic distance factor). Positive (0 = negative interaction, 1 = positive interaction) = effect of positive intergroup contact compared to negative (Valence factor).

**Table S27. The moderation of Curiosity in the models for MARS and RAT, Study 2**

|  | ***MARS*** | | | | | ***RAT*** | | | | |
| --- | --- | --- | --- | --- | --- | --- | --- | --- | --- | --- |
|  | *b* | *SE* | β | *95% CI* | *𝑝* | *b* | *SE* | β | *95% CI* | *𝑝* |
| Intercept | 3.73 | 0.59 | .00 | 2.57 – 4.90 | <.001 | 1.59 | 0.70 | .00 | 0.22 – 2.96 | .023 |
| Atypical | 0.08 | 0.83 | -.04 | -1.54 – 1.71 | .922 | -1.02 | 0.98 | -.09 | -2.94 – 0.89 | .295 |
| Positive | -0.36 | 0.83 | -.04 | -1.98 – 1.26 | .661 | -1.29 | 0.97 | .04 | -3.21 – 0.62 | .184 |
| Curiosity | -0.03 | 0.11 | -.01 | -0.25 – 0.20 | .822 | 0.08 | 0.13 | .13 | -0.18 – 0.34 | .557 |
| Atypical × Positive | -0.14 | 1.20 | .01 | -2.49 – 2.22 | .909 | 1.66 | 1.41 | -.02 | -1.11 – 4.43 | .240 |
| Atypical × Curiosity | -0.04 | 0.16 | -.01 | -0.35 – 0.27 | .793 | 0.15 | 0.18 | -.01 | -0.21 – 0.52 | .405 |
| Positive × Curiosity | 0.04 | 0.16 | .02 | -0.27 – 0.35 | .790 | 0.28 | 0.19 | .03 | -0.09 – 0.65 | .133 |
| Atypical × Positive × Curiosity | 0.04 | 0.23 | .01 | -0.41 – 0.49 | .864 | -0.34 | 0.27 | -.05 | -0.87 – 0.19 | .206 |
| R^2^ | 0.004 | | | | | 0.030 | | | | |

Note. Curiosity = a measure of individuals’ desire to acquire knowledge and explore new ideas. Atypical (0 = typical interaction, 1 = atypical interaction) = effect of atypical outgroup member compared to prototypical (Semantic distance factor). Positive (0 = negative interaction, 1 = positive interaction) = effect of positive intergroup contact compared to negative (Valence factor).

**Table S28. The moderation of Curiosity in the models for Cultural Deprovincialization, Group Deprovincialization, and Environmental concern, Study 2**

|  | ***Cultural Deprovincialization*** | | | | | ***Group Deprovincialization*** | | | | | ***Environmental concern*** | | | | |
| --- | --- | --- | --- | --- | --- | --- | --- | --- | --- | --- | --- | --- | --- | --- | --- |
|  | *b* | *SE* | β | *95% CI* | *𝑝* | *b* | *SE* | β | *95% CI* | *𝑝* | *b* | *SE* | β | *95% CI* | *𝑝* |
| Intercept | 4.08 | 0.42 | .00 | 3.26 – 4.91 | <.001 | 5.11 | 0.39 | -.00 | 4.35 – 5.87 | <.001 | 5.19 | 0.41 | -.00 | 4.37 – 6.00 | <.001 |
| Atypical | -0.94 | 0.59 | -.08 | -2.10 – 0.21 | .108 | -0.76 | 0.54 | -.14 | -1.82 – 0.29 | .156 | -0.13 | 0.58 | -.05 | -1.27 – 1.01 | .820 |
| Positive | 0.12 | 0.59 | .03 | -1.03 – 1.27 | .841 | -0.11 | 0.54 | -.00 | -1.16 – 0.95 | .843 | 1.12 | 0.58 | -.01 | -0.02 – 2.25 | .054 |
| Curiosity | 0.37 | 0.08 | .37 | 0.22 – 0.53 | <.001 | 0.24 | 0.07 | .32 | 0.10 – 0.39 | .001 | 0.19 | 0.08 | .10 | 0.03 – 0.35 | .017 |
| Atypical × Positive | 0.97 | 0.85 | .10 | -0.69 – 2.64 | .252 | 0.09 | 0.78 | -.00 | -1.44 – 1.62 | .906 | -0.37 | 0.84 | .02 | -2.02 – 1.28 | .661 |
| Atypical × Curiosity | 0.12 | 0.11 | .03 | -0.10 – 0.34 | .286 | 0.10 | 0.10 | .05 | -0.10 – 0.30 | .321 | -0.00 | 0.11 | .02 | -0.22 – 0.21 | .993 |
| Positive × Curiosity | -0.05 | 0.11 | -.05 | -0.27 – 0.17 | .666 | 0.02 | 0.10 | .01 | -0.18 – 0.22 | .834 | -0.23 | 0.11 | -.10 | -0.44 – -0.01 | .041 |
| Atypical × Positive × Curiosity | -0.12 | 0.16 | -.03 | -0.43 – 0.20 | .468 | -0.02 | 0.15 | -.01 | -0.31 – 0.27 | .890 | 0.09 | 0.16 | .02 | -0.23 – 0.40 | .582 |
| R^2^ | 0.153 | | | | | 0.120 | | | | | 0.024 | | | | |

Note. Curiosity = a measure of individuals’ desire to acquire knowledge and explore new ideas. Atypical (0 = typical interaction, 1 = atypical interaction) = effect of atypical outgroup member compared to prototypical (Semantic distance factor). Positive (0 = negative interaction, 1 = positive interaction) = effect of positive intergroup contact compared to negative (Valence factor).

**Table S29. The moderation of Curiosity in the models for AUT dimensions, Study 2**

|  | ***Fluency - AUT*** | | | | | ***Elaboration - AUT*** | | | | |
| --- | --- | --- | --- | --- | --- | --- | --- | --- | --- | --- |
|  | *b* | *SE* | β | *95% CI* | *𝑝* | *b* | *SE* | β | *95% CI* | *𝑝* |
| Intercept | 5.22 | 1.84 | -.00 | 1.61 – 8.84 | .005 | 5.17 | 2.27 | -.00 | 0.70 – 9.63 | .023 |
| Atypical | -1.84 | 2.57 | -.06 | -6.89 – 3.20 | .474 | -0.02 | 3.16 | -.06 | -6.23 – 6.18 | .994 |
| Positive | 0.16 | 2.56 | -.01 | -4.88 – 5.20 | .951 | 2.30 | 3.15 | -.02 | -3.88 – 8.49 | .465 |
| Curiosity | 0.55 | 0.35 | .11 | -0.14 – 1.24 | .117 | 0.80 | 0.43 | .10 | -0.05 – 1.65 | .066 |
| Atypical × Positive | 3.89 | 3.72 | -.00 | -3.41 – 11.20 | .296 | -0.04 | 4.56 | -.02 | -8.99 – 8.91 | .993 |
| Atypical × Curiosity | 0.28 | 0.49 | -.01 | -0.68 – 1.23 | .571 | -0.06 | 0.60 | -.01 | -1.23 – 1.11 | .920 |
| Positive × Curiosity | -0.05 | 0.49 | -.05 | -1.01 – 0.92 | .926 | -0.44 | 0.60 | -.05 | -1.62 – 0.75 | .467 |
| Atypical × Positive × Curiosity | -0.76 | 0.71 | -.05 | -2.15 – 0.63 | .282 | -0.07 | 0.87 | -.00 | -1.77 – 1.63 | .935 |
| R^2^ | 0.021 | | | | | 0.01 | | | | |

Note. Curiosity = a measure of individuals’ desire to acquire knowledge and explore new ideas. Atypical (0 = typical interaction, 1 = atypical interaction) = effect of atypical outgroup member compared to prototypical (Semantic distance factor). Positive (0 = negative interaction, 1 = positive interaction) = effect of positive intergroup contact compared to negative (Valence factor).

**Table S29 (continued). The moderation of Curiosity in the models for AUT dimensions, Study 2**

|  | ***Flexibility - AUT*** | | | | | ***Originality - AUT*** | | | | |
| --- | --- | --- | --- | --- | --- | --- | --- | --- | --- | --- |
|  | *b* | *SE* | β | *95% CI* | *𝑝* | *b* | *SE* | β | *95% CI* | *𝑝* |
| Intercept | 3.91 | 1.22 | -.00 | 1.52 – 6.30 | .001 | 0.06 | 0.33 | -.00 | -0.57 – 0.70 | .843 |
| Atypical | -1.58 | 1.70 | -.09 | -4.91 – 1.75 | .352 | -0.31 | 0.46 | -.04 | -1.20 – 0.59 | .501 |
| Positive | 0.61 | 1.69 | -.04 | -2.71 – 3.94 | .718 | 0.53 | 0.45 | -.01 | -0.37 – 1.42 | .247 |
| Curiosity | 0.30 | 0.23 | .09 | -0.16 – 0.75 | .202 | 0.07 | 0.06 | .06 | -0.06 – 0.19 | .286 |
| Atypical × Positive | 1.36 | 2.46 | .03 | -3.46 – 6.19 | .580 | -0.08 | 0.66 | -.03 | -1.38 – 1.21 | .899 |
| Atypical × Curiosity | 0.18 | 0.32 | .01 | -0.45 – 0.81 | .580 | 0.06 | 0.09 | .04 | -0.11 – 0.23 | .507 |
| Positive × Curiosity | -0.19 | 0.32 | -.05 | -0.83 – 0.45 | .556 | -0.10 | 0.09 | -.06 | -0.27 – 0.08 | .271 |
| Atypical × Positive × Curiosity | -0.19 | 0.47 | -.02 | -1.11 – 0.72 | .677 | -0.00 | 0.13 | -.00 | -0.25 – 0.25 | .996 |
| R^2^ | 0.021 | | | | | 0.013 | | | | |

Note: Curiosity = a measure of individuals’ desire to acquire knowledge and explore new ideas. Atypical (0 = typical interaction, 1 = atypical interaction) = effect of atypical outgroup member compared to prototypical (Semantic distance factor). Positive (0 = negative interaction, 1 = positive interaction) = effect of positive intergroup contact compared to negative (Valence factor).
